# Natural compounds as angiogenic enzyme thymidine phosphorylase inhibitors: *In vitro* biochemical inhibition, mechanistic, and *in silico* modeling studies

**DOI:** 10.1371/journal.pone.0225056

**Published:** 2019-11-19

**Authors:** Sumaira Javaid, Muniza Shaikh, Narjis Fatima, M. Iqbal Choudhary

**Affiliations:** 1 Dr. Panjwani Center for Molecular Medicine and Drug Research, International Center of Chemical and Biological Sciences, University of Karachi, Karachi, Pakistan; 2 H. E. J. Research Institute of Chemistry, International Center for Chemical and Biological Sciences, University of Karachi, Karachi, Pakistan; 3 Department of Biochemistry, Faculty of Sciences, King Abdulaziz University, Jeddah, Saudi Arabia; Aligarh Muslim University, INDIA

## Abstract

Natural flora is the richest source of novel therapeutic agents due to their immense chemical diversity and novel biological properties. In this regard, eighteen natural products belonging to different chemical classes were evaluated for their thymidine phosphorylase (TP) inhibitory activity. TP shares identity with an angiogenic protein platelet derived endothelial cell growth factor (PD-ECGF). It assists tumor angiogenesis and is a key player in cancer progression, thus an ideal target to develop anti-angiogenic drugs. Eleven compounds **1**–**2**, **5**–**10**, **11**, **15**, and **18** showed a good to weak TP inhibitory activity (IC_50_ values between 44.0 to 420.3 μM), as compared to standards *i*.*e*. tipiracil (IC_50_ = 0.014 ± 0.002 *μ*M) and 7-deazaxanthine (IC_50_ = 41.0 ± 1.63 μM). Kinetic studies were also performed on active compounds, in order to deduce the mechanism of ligand binding to enzyme. To get further insight into receptor protein (enzyme) and ligand interaction at atomic level, *in- sillico* studies were also performed. Active compounds were finally evaluated for cytotoxicity test against mouse fibroblast (3T3) cell line. Compound **18** (Masoprocol) showed a significant TP inhibitory activity (IC_50_ = 44.0 ± 0.5 μM). Kinetic studies showed that it inhibits the enzyme in a competitive manner (*K*i = 25.6 ± 0.008 μM), while it adopts a binding pose different than the substrate thymidine. It is further found to be non-toxic in MTT cytotoxicity assay. This is the first report on TP inhibitory activity of several natural compounds, some of which may serve as leads for further research towards drug the development.

## Introduction

Cancer is one of the primary causes of death worldwide, with approximately one in six person dies because of some malignant conditions. In the last few decades, early diagnosis and treatment have improved the patient’s life quality, and survival rates. However, there are still high rates of recurrence, invasiveness, and metastases. To improve efficacy, new therapeutic interventions are required which target different and important stages of tumor progression. In this context, angiogenesis has emerged as a hallmark of tumor growth. Since the last five decades, it has been hypothesized that blocking angiogenesis could be an effective way to combat the disease’s progression. Thenceforth, a large number of molecules, targeting the process of angiogenesis, have been under pre-clinical and clinical trials. Some of these molecules are already approved as drugs by the U.S. Food and Drug Administration (FDA). Thymidine phosphorylase (TP) is among the several factors that stimulate the growth of blood vessels, and it described as a validated target for the antiangiogenic compounds development [1].

Enzyme thymidine phosphorylase (TP) is involved in pyrimidine salvage pathway, regulating the nucleotide homeostasis which is required for DNA repair and replication. It catalyzes the reversible phosphorolysis of thymidine to thymine and 2-deoxyribose-1-phosphate. 2-Deoxyribose-1-phosphate then undergoes dephosphorylation to 2-deoxyribose, which actually triggers tumor angiogenesis. 2-Deoxyribose affects endothelial-cell migration by activating the downstream integrin signaling pathway. It also augment the expression and/or release of several angiogenic growth factors, such as VEGF, MMPs, cytokines, interleukins, and others, which in the tumor microenvironment enhance the angiogenesis, and cancer metastasis. TP is reported to share sequence homology with an angiogenic growth factor (Platelet derived endothelial cell growth factor; PD-ECGF), which promotes angiogenesis by facilitating endothelial cells proliferation, and migration [2, 3].

Over-expression of TP has been associated with cancer aggressiveness, and poor prognosis. Different solid tumors, including breast, colorectal, bladder, and esophageal cancers are being reported with high levels of TP [4–7]. In addition to this some other diseases, such as rheumatoid arthritis, psoriasis, and inflammation also show high levels of TP activity [8]. Several TP inhibitors have been reported with potent activities *in vitro*, however, only one TP inhibitor (tipiracil) in combination of a cytotoxin (trifluridine) is US-FDA approved for the management of colorectal cancer. Usage of this combination drug (Lonsruf) is compromised with several side effects, such as neutropenia, anemia, myelosuppression, etc [9–20].

Our research group has previously reported several synthetic and few natural inhibitors of TP [21–26]. In the present study, an attempt was made to screen selected natural compounds for their TP inhibitory potential. Natural compounds present a long-standing tradition as valuable starting points in drug discovery program. Structural diversity makes them privileged sources for chemical probes. Medicinal plants are rich source of producing natural products and many of these are used as active ingredient of modern medicine [27–28]. Only less than 10% of the natural flora is investigated so far, therefore, nature holds a great promise for discovery of leads against most common, and emerging diseases [29].

**Fig 1:**
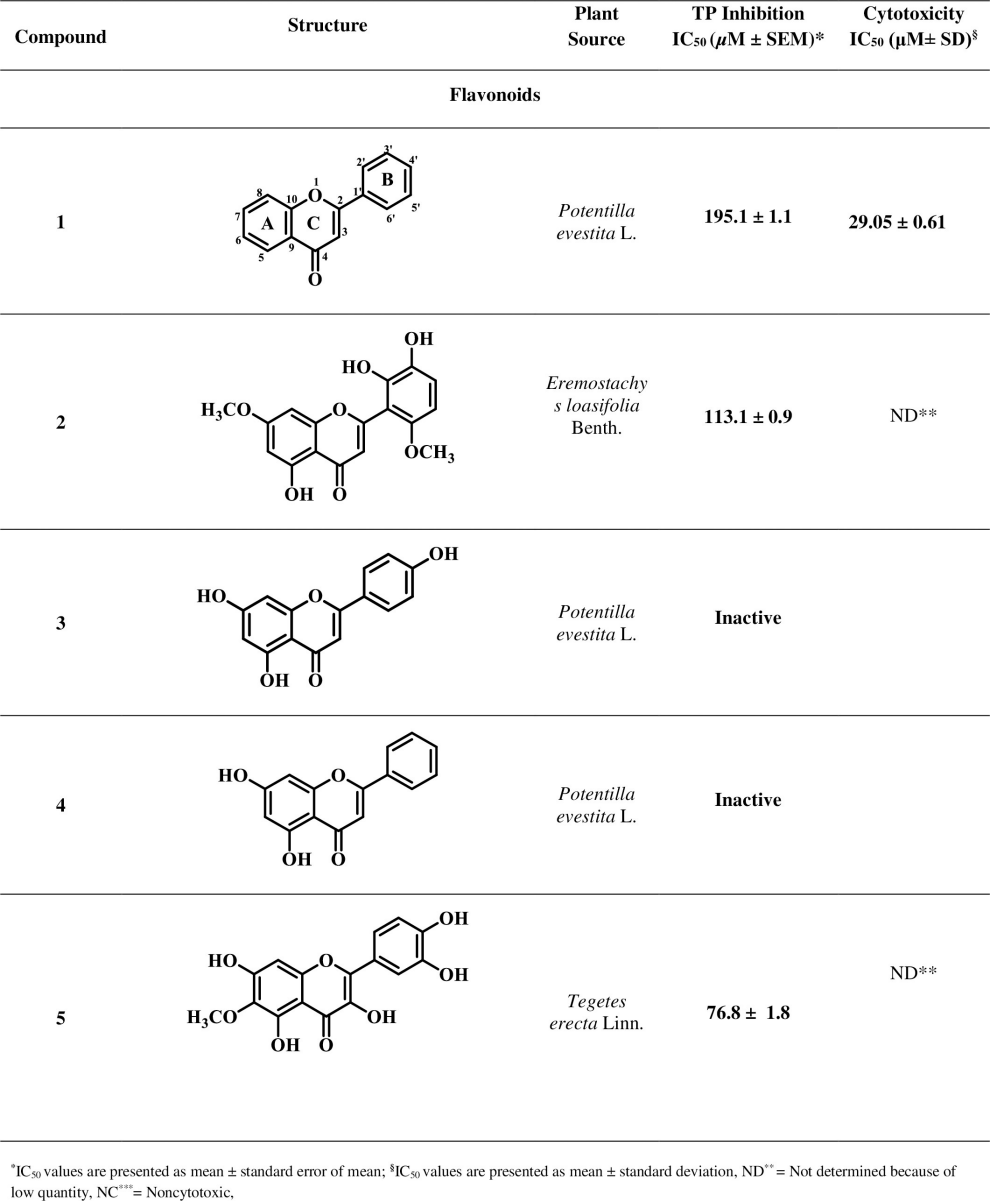
Thymidine Phosphorylase Inhibitory Activity of Compounds 1–18.

**Fig 2:**
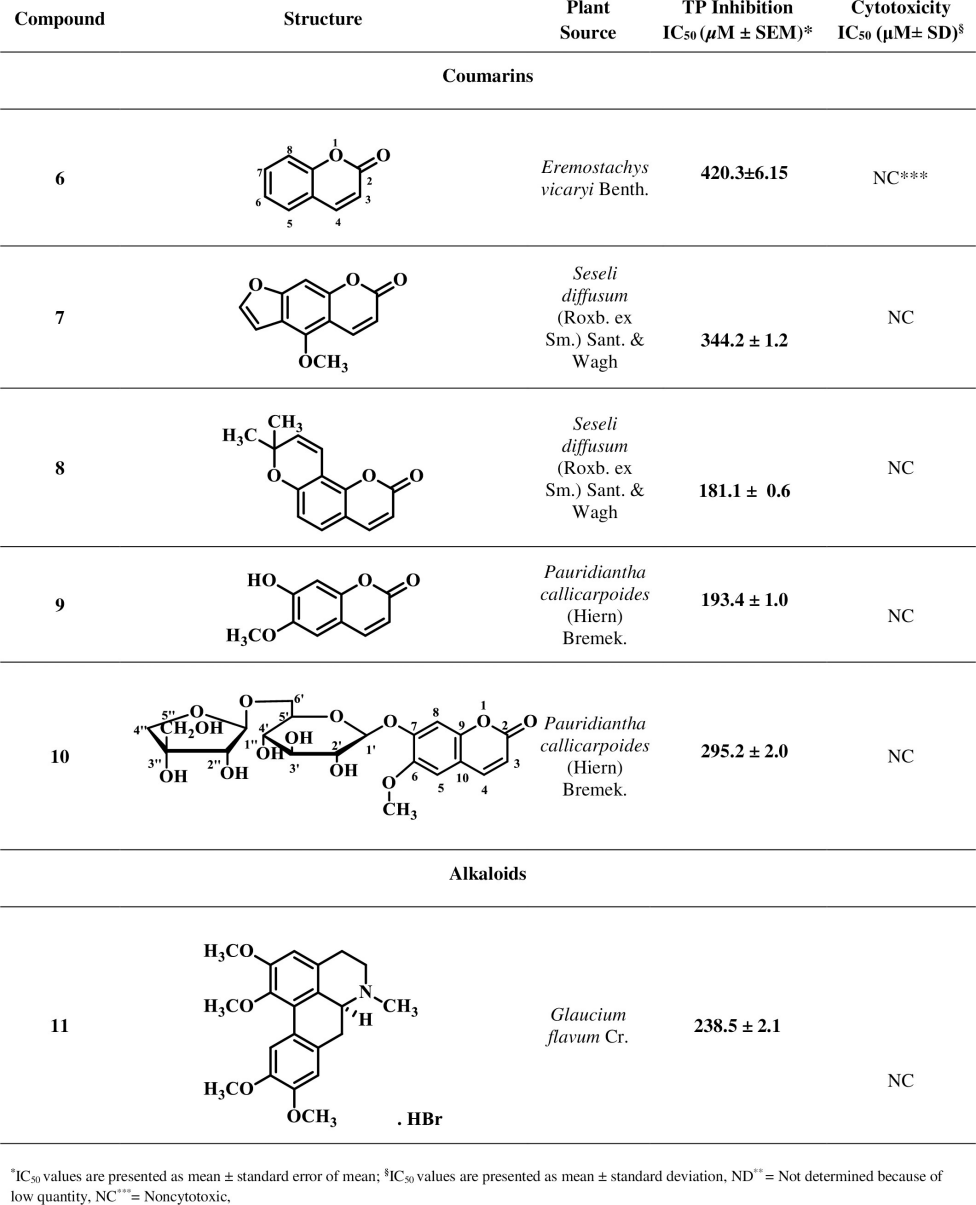
Thymidine Phosphorylase Inhibitory Activity of Compounds 1–18 (Continued).

**Fig 3:**
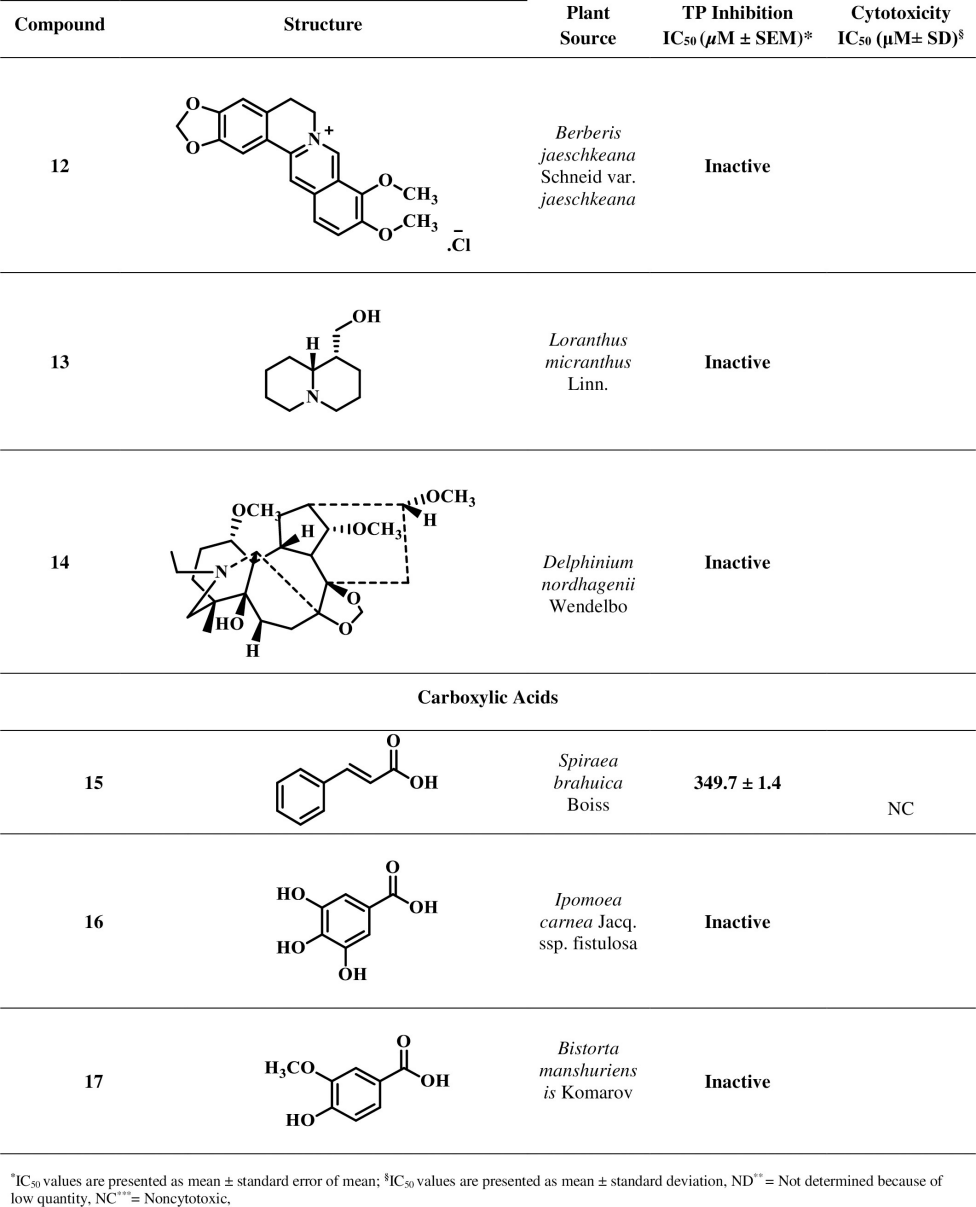
Thymidine Phosphorylase Inhibitory Activity of Compounds 1–18 (Continued).

**Fig 4:**
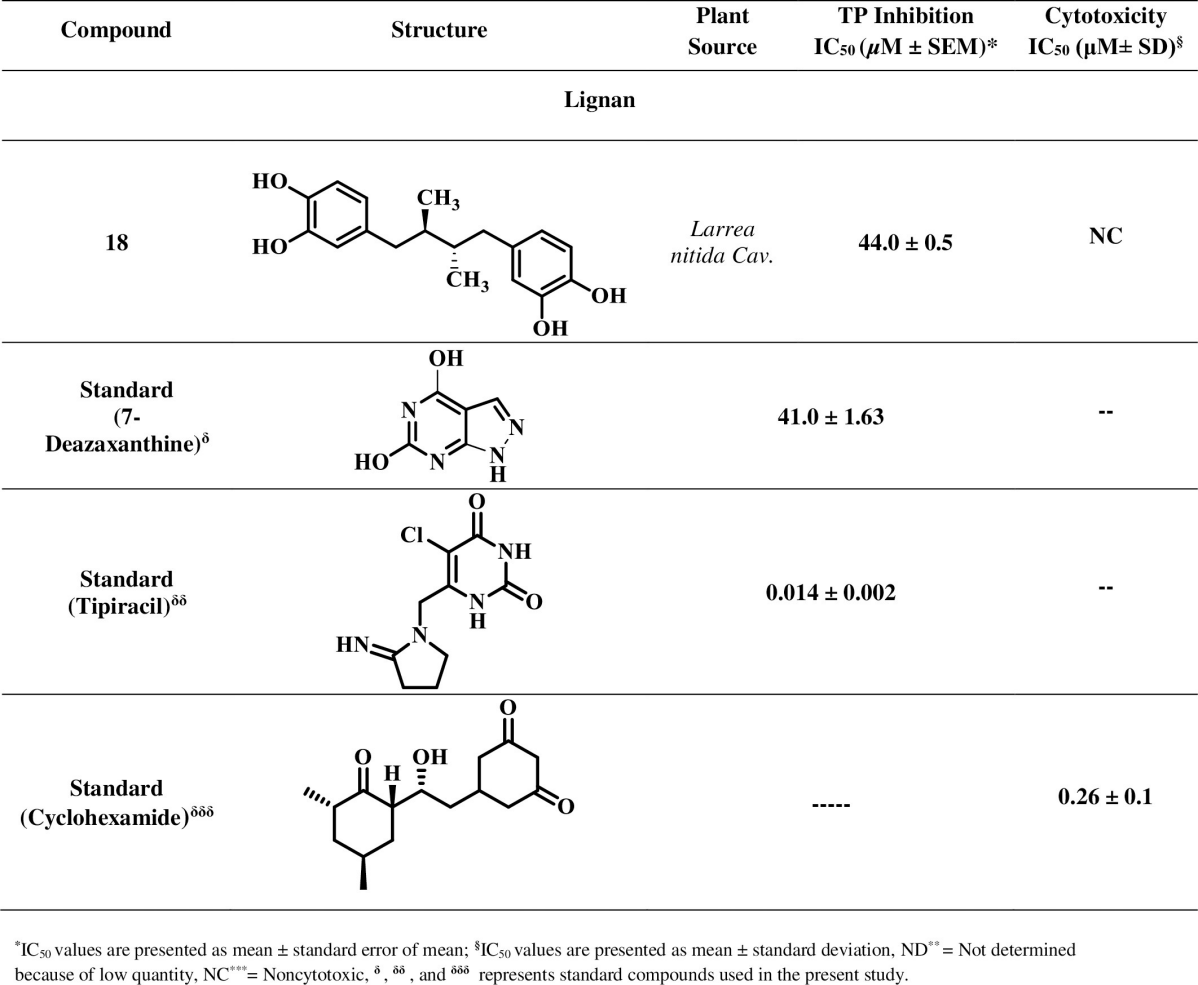
Thymidine Phosphorylase Inhibitory Activity of Compounds 1–18 (Continued).

Based on reported medicinal importance particularly the anti-cancer activity, some natural compounds belonging to class coumarin, flavonoid, carboxylic acid, alkaloid, and lignan were selected randomly and evaluated for their TP inhibitory activity [30–37]. These natural compounds are not reported earlier for the TP inhibitory activity. Isolation and spectroscopic data for the compounds are presented in the S1 Table. The current study led to the identification of new potential TP inhibitors, as summarized in Figs 1–4.

## Materials and methods

Enzyme thymidine phosphorylase (*E*. *coli*, E.C. No. 2.4.2.4, CAS # 9030-23-3), DMEM (Dulbecco's modified eagle medium, CAT # SLM-241), and cycloheximide (CAS # 66-81-9) were obtained from Sigma Aldrich, USA. DMSO (Dimethylsulfoxide, CAS # 67-68-5), and standard compounds (tipiracil; CAS # 183204-72-0, 7-deazaxanthine; CAS # 39929-79-8) were obtained from Fisher Scientific, Germany, and Santa Cruz Biotechnology Inc. (USA), respectively. K_2_HPO_4_ (Dipotassium hydrogen phosphate; CAS # 7758-11-4), and KH_2_PO_4_ (Potassium dihydrogen phosphate; CAS # 7778-77-0) were obtained from Merck, Germany. Mouse fibroblast cell line (3T-3, CAT # ATCC^®^ CRL-1658^™^), 0.25% Trypsin EDTA (CAS # 25200056), FBS (fetal bovine serum; CAT # FBS-ES-22A), MTT (3-(4,5-dimethythiazol-2-yl)-2,5-diphenyl tetrazolium bromide, CAS # 298-93-1), and 0.4% Trypan Blue solution (CAS # K940) were obtained from American Type Culture Collection, USA, Gibco, Invitrogen, New Zealand, A&E Scientific, USA, MP Biomedicals, France, and Amersco, USA, respectively.

### Source of natural compounds

#### Flavonoids

Compound **1** (CAS No. 525-82-6; purity ≥ 99% as indicated by HPLC), originally isolated from *Potentilla evestita* L., was purchased from Sigma Aldrich, USA. Compounds **2**, **3**, **4**, and **5** were isolated from *Eremostachys loasifolia* Benth., *Potentilla evestita* L., and *Tagetes patula* Linn., respectively [38–40]. These compounds **2**–**4** were > 99% pure as assayed by HPLC techniques (See S1 Information and S1 Table for details about extraction and spectroscopic data).

#### Coumarins

Compound **6** was isolated from *Eremostachys vicaryi* Benth. Compounds **7**–**8** were isolated from *Seseli diffusum* (Roxb. ex Sm.) Sant. & Wagh, while **9**–**10** were isolated from *Pauridiantha callicarpoides* (Hiern) Bremek. These compounds were > 99% pure as assayed by HPLC techniques [41–43] (See S1 Information and S1 Table for details about extraction and spectroscopic data).

#### Alkaloids

Compounds **11** (Glaucine HBr, LOT No. 00007241–807; purity ≥ 94.9% from HPLC) and **12** (Berberine chloride, CAS No. 633-65-8; purity > 98% from TLC) isolated from *Glaucium flavum* Cranz. and *Berberis jaeschkeana* Schneid var. *jaeschkeana* respectively, were obtained from ChromaDex (Irvine, California, USA). Compound **13** (Lupinine, CAS No. 486-70-4; purity ≥ 97% from HPLC), isolated from *Loranthus micranthus* Linn. was bought from Santa Cruze Biotechnolgy Inc., USA, for the present study. Compound **14** (Nordhagenine A) was isolated from *Delphinium nordhagenii* Wendelbo [44] (See S1 Information and S1 Table for details about extraction and spectroscopic data).

#### Carboxylic acids

Compounds **15** (Cinnamic acid, CAS No. 140-10-3; purity ≥ 97% by titration with NaOH), **16** (Gallic acid, CAS No. 149-91-7; purity ≥ 97% by titration with NaOH), and **17** (Vanillic acid, CAS No. 121-34-6; purity ≥ 97% by titration with NaOH), originally isolated from *Spiraea brahuica* Boiss, *Ipomoea carnea* Jacq. ssp. fistulosa, and *Bistorta manshuriensis* Komarov. respectively, were purchased from Sigma Aldrich, USA (See S1 Information and S1 Table for details about extraction and spectroscopic data).

#### Lignan

Compound **18** (Masoprocol, CAS Number 500-38-9; purity ≥ 90% purity as assayed by HPLC) was purchased from Sigma Aldrich, USA. It was originally isolated from *Larrea nitida Cav*., (See S1 Information and S1 Table for details about extraction and spectroscopic data).

#### Thymidine phosphorylase inhibition assay

In the current study, we have used commercially available recombinant *E*. *coli* TP enzyme, as human TP is not easily accessible. Substantial similarities in terms of structural and active site residues exist between *E*. *coli* and mammalian TP enzymes, therefore *E*. *coli* TP generally serves as a primary model for the identification of lead inhibitors of TP [3].

Thymidine phosphorylase inhibition assay was carried out spectrophotometrically [45]. Briefly, 0.058 U of TP enzyme (E.C. No. 2.4.2.4, *E*. *coli*) was incubated with 500 μM of test compounds at 30°C for 10 minutes. After that 1.5 mM of substrate thymidine was added to 96-well plate, and changes in absorbance were monitored for 10 minutes at 290 nm in microtiter plate reader (Spectramax, M5, Molecular Devices, CA, USA). Enzyme and substrate solution were prepared in 50 mM phosphate buffer of pH 7.0, while test compounds were prepared in DMSO (final concentration 4.5%). Tipiracil and 7-deazaxanthine were used as standard inhibitors, and each experiment was performed in triplicate.

#### Mechanistic studies

In mechanistic studies, TP enzyme was incubated with different concentrations of inhibitors at 30°C for 10 min. Reaction was then initiated by adding four different concentrations of substrate, *i*.*e*. thymidine (0.1875–1.5 mM). Enzyme activity was measured under steady state conditions by measuring changes in absorbance for another 10 min at 30°C at 290 nm, on microtiter plate reader (Spectramax, M5, Molecular Devices, CA, USA). Every experiment is performed in triplicate.

### Molecular docking studies

#### Ligand preparation

Prior to docking, the molecules (ligands) were processed *via Ligprep* module in Maestro Schrӧdinger 10.5. It involves the generation of low energy 3-D structures from 2-D structures of compounds in SD format. Possible ionization states and correct protonation were generated using *Epik* module which predict the tautomeric state, and generate energetic penalties for each molecule conformation it predicts [46].

#### Protein preparation

X-Ray crystallographic structure of *E*. *coli* TP was used for docking studies (PDB ID: 4LHM). Maestro Schrӧdinger software was used to prepared protein by employing the *Protein Preparation Wizard* 10.5 [47, 48]. OPLS-2005 force field was used to add missing hydrogens, and for the assignment of partial charges. Optimization of heavy atoms and hydrogens was then carried out by subjecting the structure to restrained minimization. The co-crystallized water molecules were retained because they were present in the active site, involving the formation of general hydrogen bond network. Since the sulfate ion was replaced with phosphate, it occupied the same place in active site in crystal structure as that of phosphate ion.

#### Searching for allosteric binding sites and molecular docking analysis

To find out the allosteric site for non-competitive and uncompetitive inhibitors, site recognition software SiteMap 3.7 [49, 50] Maestro version 10.5 from Schrödinger was run on crystal structure to identify the top 5 ranked potential ligand-binding pockets.

The grid box with dimensions of 15Ǻ x 15Ǻ x 15Ǻ was defined to confine the mass of centre of each docked ligand. Extra precision (XP) mode of Glide based on OPLS-2005 force field was run for rigid receptor docking protocol [51–54]. Molecular mechanics-generalized Born surface area (MM-GBSA) method in Prime was used for rescoring the docked pose of ligand [55]. These poses were taken as inputs for the energy minimization of the protein–ligand complexes (E_complex_), the free protein (E_receptor_), and the free ligands (E_ligand_). The binding free energy ΔG_bind_ was determined according to the following equation:

$${\Delta G}_{bind}\;=\;E{\;}_{complex\;\left(minimized\right)}-E{\;}_{ligand\;\left(minimized\right)\;}-E{\;}_{receptor\;\left(minimized\right)\;}$$

### *In vitro* cytotoxicity assay

Cytotoxicity of active compounds was evaluated spectrophotometrically by standard MTT (3-[4,5-dimethylthiazole-2-yl]-2,5-diphenyl-tetrazolium bromide) assay, following the method of Demas *et al*. In cytotoxicity assay, reduction of MTT dye to formazan by mitochondrial enzyme (succcinate dehydogenase) is measured. The reduction of MTT can only occur in metabolically active cell, so the enzyme activity is actually a measure of cell viability [24, 56].

Briefly, the mouse fibroblast cells (3T3) were cultured in DMEM media containing 5% FBS, 100 IU/ mL penicillin, and 100 μg/ mL streptomycin. Cell suspension (5×10^4^cells/ mL) was dispensed in flat-bottomed 96-well plates, and incubated at 37°C and with 5% CO_2_. After the overnight incubation, old media was aspirated and fresh media containing different concentrations of compounds (prepared in DMSO) were added, and plate was further incubated for 48 hrs. After that, MTT dye (0.5 mg/ mL) was added into each well, and the plate was further incubated for 4 hrs. Following this, DMSO (100 μL) was added into each well, and the level of MTT reduction to formazan with in the cells was calculated by taking the absorbance at 540 nm using a micro plate reader (Spectra Max plus, Molecular Devices, CA, USA).

### Analysis of the experiments

#### *In vitro* TP spectrophotometric assay

Enzyme activity was measured under steady-state conditions by observing changes in absorbance for 10 min (at 290 nm) on microtiter plate reader (Spectra Max M5, Molecular Devices, CA, USA). Changes in absorbance upon addition of test compounds were analyzed with the SoftMax Pro 4.8 software which was purchased from Molecular Devices, CA, USA. To calculate the percent inhibition, following formula was used:

$$\%\;Inhibition\;=\;100-\frac{Absorbance\;of\;test\;compound}{Absorbance\;of\;control}\times 100$$

Inhibitory potential of test compounds is represented in Figs 1–4 as IC_50_ value (Inhibitory concentration). The IC_50_ represents concentration of compounds that inhibit the degradation of thymidine to thymine by 50%. It was calculated by measuring the effects of different concentrations of inhibitors on the degradation of thymidine. EZ-Fit, Enzyme kinetics software (purchased from Perrella Scientific, Inc., USA) was used to deduce the IC_50_ values.

### Analysis of mechanistic assay

Mechanistic studies were performed to find out the mechanism of inhibition of active compounds. Lineweaver-Burk plot was used to determine the type of inhibition, where the reciprocal of rate of the reaction were plotted against the reciprocal of substrate concentration. Dissociation constant (*K*_*i*_) were determined by secondary re-plot of Lineweaver-Burk plots. Kinetic data was analysed by GraFit 7 data analysis software (purchased from Erithacus Software Limited, UK) [57].

### Analysis of docking studies

To find out the allosteric site for non-competitive and uncompetitive inhibitors, site recognition software SiteMap 3.7 Maestro version 10.5 from Schrödinger was run on crystal structure to identify the top 5 ranked potential ligand-binding pockets. Molecular mechanics-generalized Born surface area (MM-GBSA) method in Prime was used for rescoring the docked pose of ligand. The binding free energy ΔG_bind_ was determined according to the–equation given earlier.

### Analysis of cytotoxicity assay

The cytotoxicity was recorded as concentration causing 50% growth inhibition (IC_50_) for 3T3 cells. Cellular viability was measured for 10 min, at 570 nm on microtiter plate reader. Changes in absorbance upon addition of test compounds were analyzed with the SoftMax Pro 4.8 software which was purchased from Molecular Devices, CA, USA. Percent inhibition was calculated using the formula mentioned earlier.

Cytotoxicity potential of test compounds is represented in Figs 1–4 as IC_50_ values. The IC_50_ represents concentration of compounds that inhibit the cell growth by 50%. It was calculated by measuring the effects of different concentrations of inhibitors on the growth of cells. EZ-Fit, Enzyme kinetics software was used to deduce the IC_50_ values.

## Results and discussion

Eighteen secondary metabolites of medicinal plant origin were evaluated for their *in vitro* TP inhibitory activity *via* biochemical method. Eleven compounds **1**, **2**, **5**–**10**, **11**, **15**, and **18** exhibited good to weak TP inhibitory activity with IC_50_ values between 44.0 to 420.3 μM, as compared to the standard inhibitor, 7-deazaxanthine (7-DX) (IC_50_ = 41.0 ± 1.63 μM). The results indicate that these compounds are effective at high micromolar concentration thus could serve as starting point for the development of new anti-TP molecules. Limited structure-activity relationship was carried out (explained below) as the current study is based on the small number of compounds.

### Structure-activity relationship of natural compounds

Among compounds **1**–**5** belonging to flavonoid class, **1**, **2**, and **5** showed a good to weak TP inhibiting ability with IC_50_ values of 76.8 to 195.1 μM, as compared to standard 7-deazaxanthine (IC_50_ = 41.0 ± 1.63 μM), while compounds **3**, and **4** were found to be inactive.

Compound **1** with a basic flavonoid skeleton showed a weak TP inhibiting activity (IC_50_ = 195.1 ± 1.1 μM). Compound **2** with OH groups at C-5, C-2', and C-3', and OCH_3_ groups at C-7 and C-6' showed a good TP inhibition (IC_50_ = 113.0 ± 0.9 μM), as compared to compound **1**. However, presence of OH groups at C-5, C-7 and C-4', as in compound **3**, and at C-5 and C-7, as in compound **4** were found unfavourable for TP inhibition. Presence of OCH_3_ at C-6 and OH at C-3, C-5, C-7, C-4', and C-5' in compound **5** enhanced the TP inhibitory potential (IC_50_ = 76.8 ± 1.1 μM). Structure-activity relationship (SAR) indicated that number and position of electron donating groups on flavonoid nucleus play role in TP inhibition. Hydroxyl and methoxy groups are expected to form hydrogen bonds and/or hydrophobic interactions with amino acid residues at active site or hydrophobic pocket of the TP enzyme.

Compounds **6**–**10** of coumarins class showed a weak TP inhibiting activity with IC_50_ values in the range of 181.1–420.3 μM, as compared to the standard 7-deazaxanthine (IC_50_ = 41.0 ± 1.63 μM) (Table 1). Among the group, compound **6** showed the weakest TP inhibition (IC_50_ = 420.3 ± 6.15 μM). Presence of furan and methoxy moieties at coumarin skeleton in compound **7** increased the inhibitory potency (IC_50_ = 344.2 ± 1.2 μM), as compared to compound **6**. TP inhibition increased further due to the presence of pyran and di-methyl groups at coumarin skeleton, as in compound **8** (IC_50_ = 181.1 ± 0.6 μM). Compound **9** with OH and OCH_3_ groups on coumarin skeleton also showed the increased TP inhibitory potential in comparison to compound **6** (IC_50_ = 193.4 ± 1.0 μM). However, replacement of hydroxyl group at C-7 with apiose glucoside moiety in compound **10** decreased the TP inhibition potency (IC_50_ = 295.2 ± 2.0 μM). This indicates that OH group is critical for inducing enzyme inhibition.

**Table 1:** Kinetic data of compounds 1, 5–11, and 18.

Compound	*Ki* ± SEM^a^ (μM)	Type of inhibition
**1**	225.0 ± 0.002	Uncompetitive
**5**	341.1 ± 0.01	Mixed
**6**	171.0 ± 0.001	Uncompetitive
**8**	162.0 ± 0.001	Uncompetitive
**9**	419.0 ± 0.001	Non-competitive
**10**	145.0 ± 0.001	Uncompetitive
**11**	180.0 ± 0.01	Uncompetitive
**18**	25.6 ± 0.008	Competitive
**7-Deazaxanthine**	45.66 ± 0.0009	Non-competitive

^a^*K*i ± SEM is dissociation constant ± Standard Error of the Mean

SAR suggested that compound **6** (coumarin) is mainly interacting with the enzyme *via* hydrophobic interaction with critical residues at the active site or hydrophobic pocket of enzyme TP. The presence of other electron donating groups (hydroxyl and methoxy) in compounds **7**–**10**, found to enhance their potential of inhibiting the TP enzyme, in comparison to compound **6**. Hydroxyl may likely to be involve in forming hydrogen bond with the amino acids at active site or hydrophobic pocket of the TP enzyme.

Among the alkaloids **11**–**14**, compound **11** exhibited a weak TP inhibitory activity (IC_50_ = 238.5 ± 2.1 μM). Methoxy groups of compound **11** proposed to interact with the enzyme’s hydrophobic pocket *via* hydrogen bonding with critical amino acid residues. Compounds **12**–**14** were found to be inactive against the TP.

Among the compounds **15**–**17** belonging to benzoic acid class, only compound **15** showed a weak TP inhibitory activity with IC_50_ of 349.7 ± 1.4 μM. This compound is likely to be engaged in hydrogen bonding with the residues present at the active site or hydrophobic pocket of the enzyme. However, the phenyl moiety can also be involved in π-π interaction with aromatic residues. Compounds **16**, and **17** were found to be inactive.

Compound **18**, a lignan, exhibited a significant TP inhibitory activity (IC_50_ = 44.0 ± 0.5 μM). Hydroxyl groups are apparently forming hydrogen bonds with critical amino acid residues of the active site.

### Mechanistic studies

**Fig 5:**
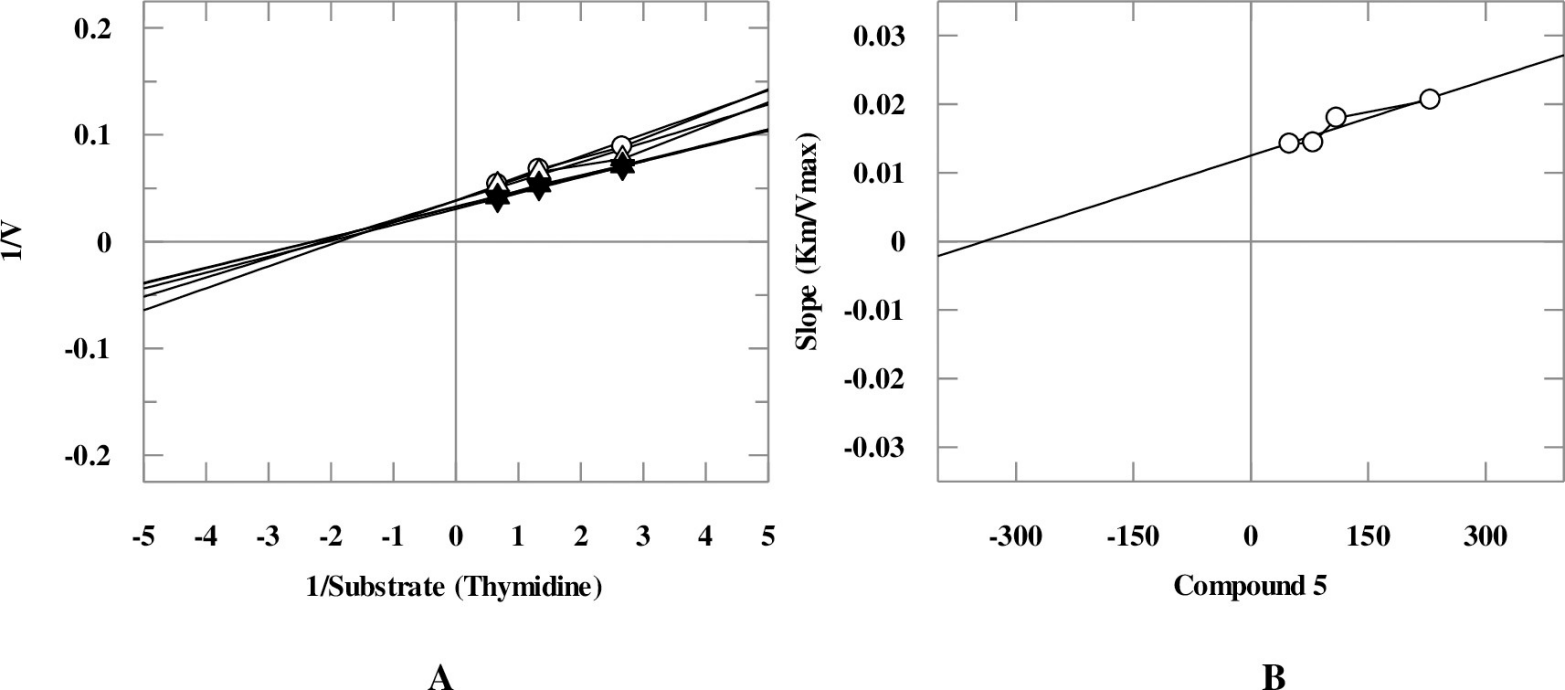
**The inhibition of TP by compound 5**, (A) is the Lineweaver-Burk plot of reciprocal rate of reaction (velocities) *verses* reciprocal of substrate (thymidine) in the absence (▼), and in the presence of 230 μM (○), 110 μM (Δ), 80 μM (▲) and 50 μM (▽) of compound **5**. (B) is secondary replot of Lineweaver-Burk plot between the slopes of each line on Lineweaver-Burk plot *versus* different concentrations of compound **5**.

**Fig 6:**
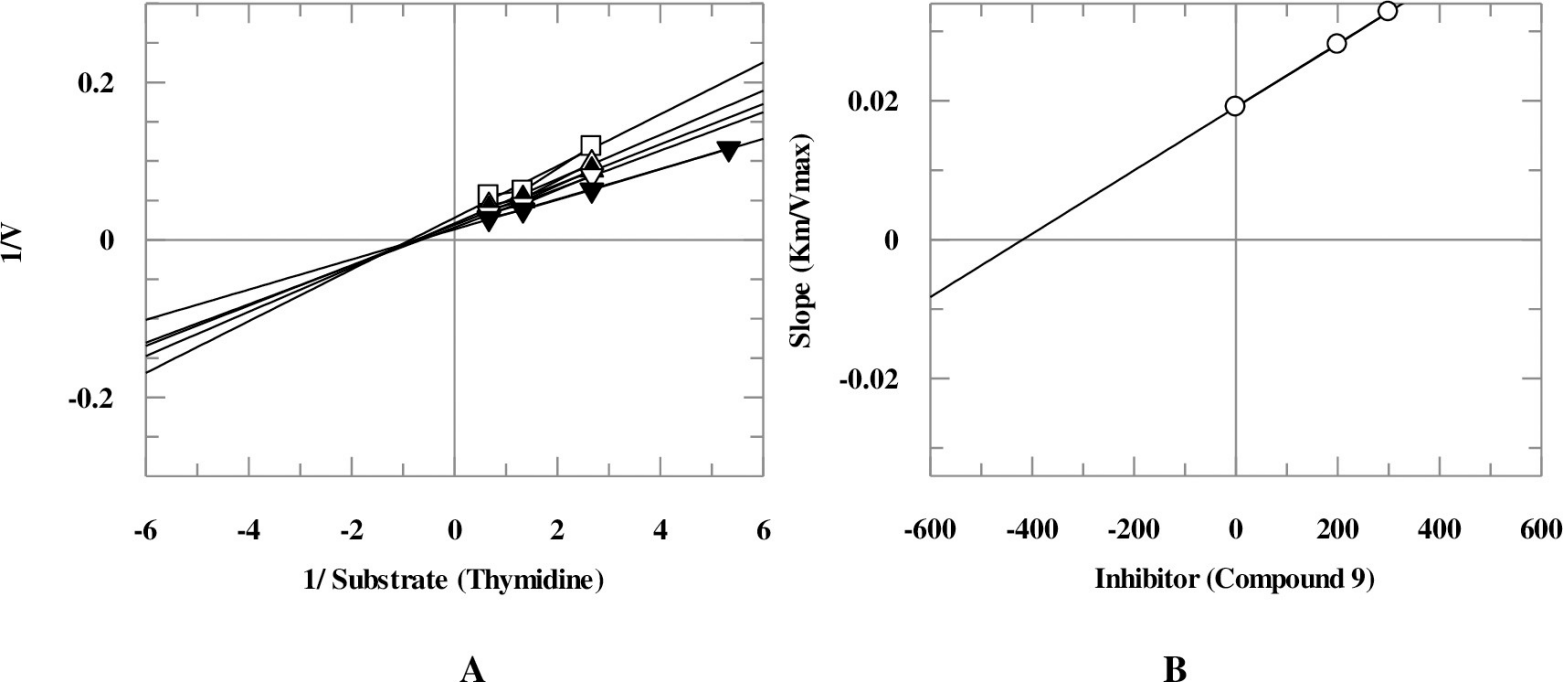
**The inhibition of TP by compound 9**, (A) is the Lineweaver-Burk plot of reciprocal rate of reaction (velocities) *verses* reciprocal of substrate (thymidine) in the absence (▼), and in the presence of 300 μM (○), 200 μM (Δ), 100 μM (▲) and 50 μM (▽) of compound **9**. (B) is secondary replot of Lineweaver-Burk plot between the slopes of each line on Lineweaver-Burk plot *versus* different concentrations of compound **9**.

**Fig 7:**
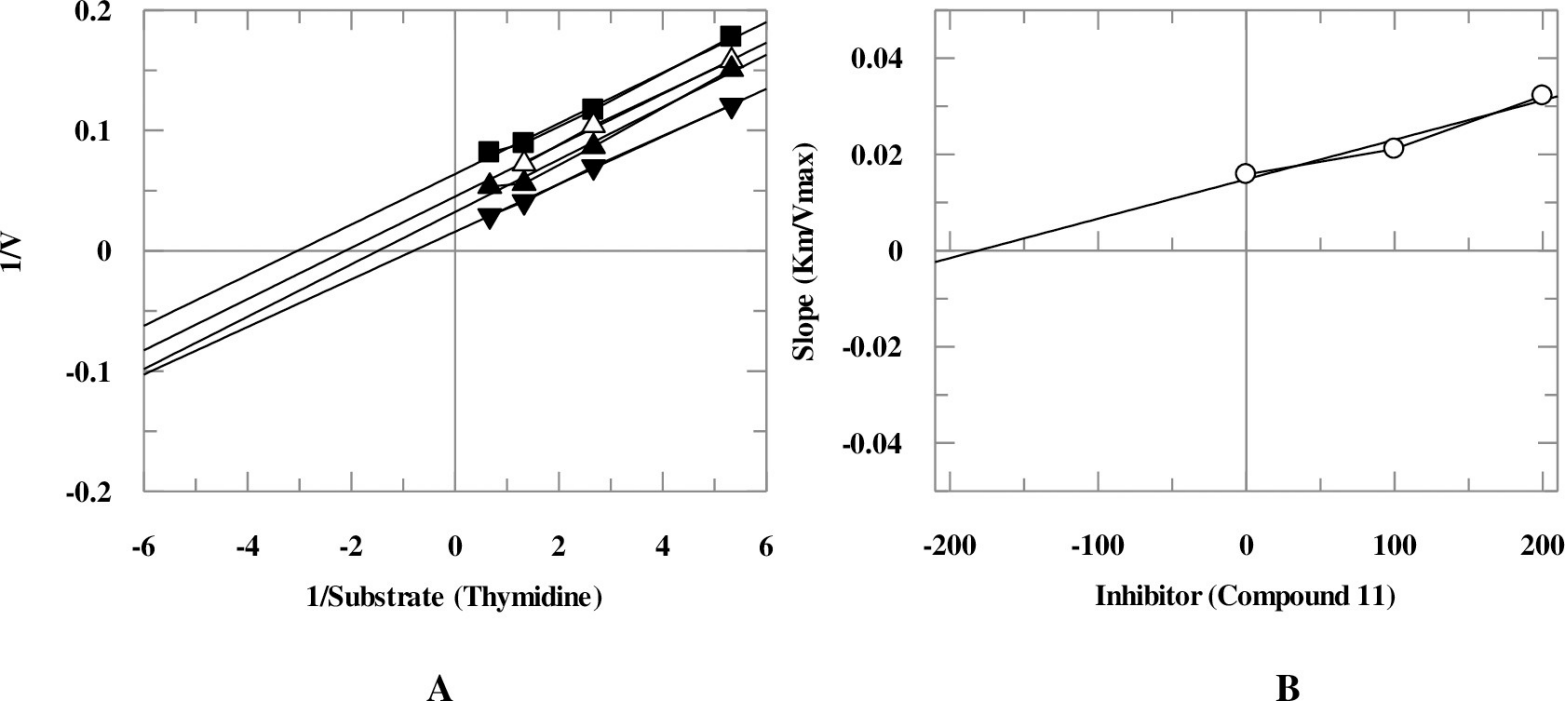
**The inhibition of TP by compound 11**, (A) is the Lineweaver-Burk plot of reciprocal rate of reaction (velocities) *verses* reciprocal of substrate (thymidine) in the absence (▼), and in the presence of 300 μM (■), 250 μM (Δ), and 200 μM (▲) compound **11**. (B) is secondary replot of Lineweaver-Burk plot between the slopes of each line on Lineweaver-Burk plot *versus* different concentrations of compound **11**.

**Fig 8:**
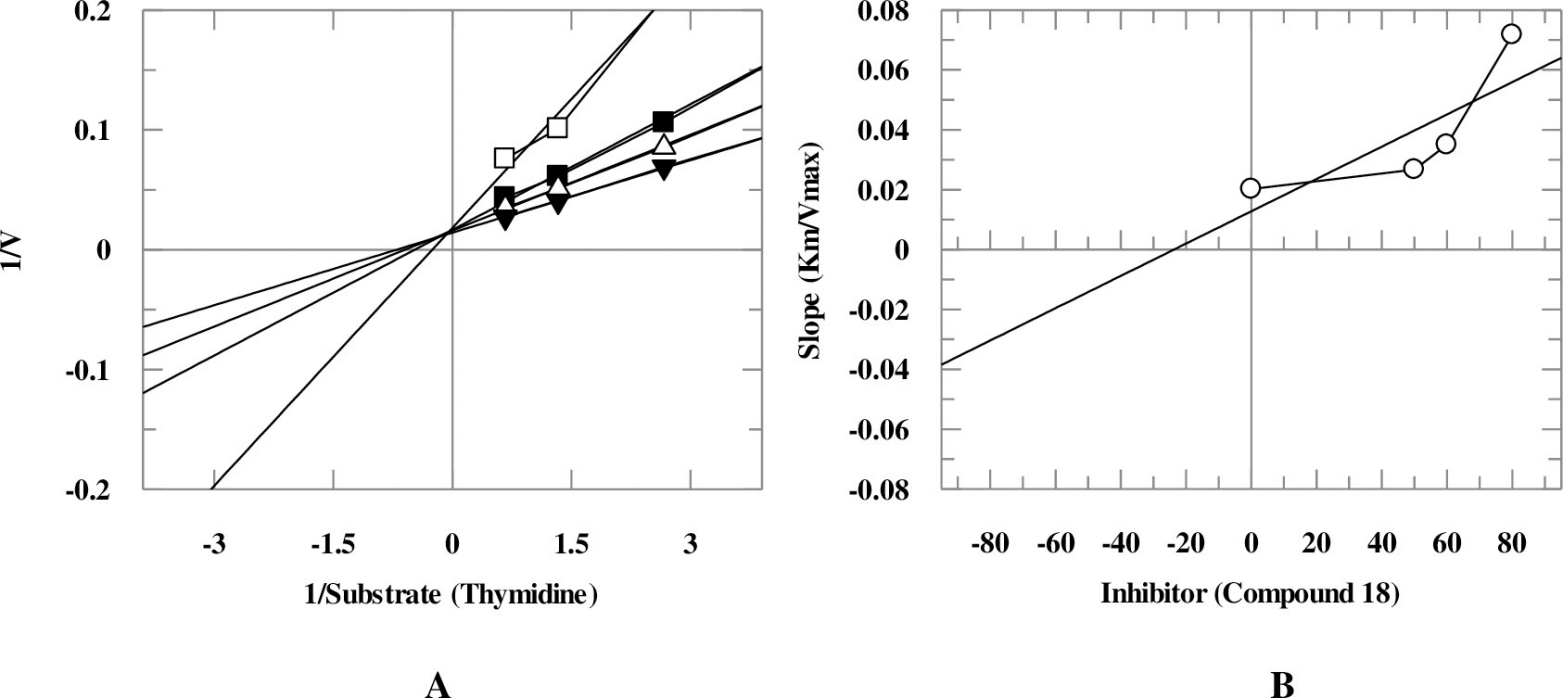
**The inhibition of TP by compound 18**, (A) is the Lineweaver-Burk plot of reciprocal rate of reaction (velocities) *verses* reciprocal of substrate (thymidine) in the absence (▼), and in the presence of 80 μM (□), 60 μM (■), and 50 μM (Δ) of compound **18**. (B) is secondary replot of Lineweaver-Burk plot between the slopes of each line on Lineweaver-Burk plot *versus* different concentrations of compound **18**.

Mechanistic studies were performed on the selected natural products. It was found that they inhibit TP enzyme in a concentration-dependent manner with dissociation constant (*K*i) values between 25.6–419.0 μM. Natural products **5**, **9**, and **18** exhibited mixed, non-competitive, and competitive modes of inhibition, respectively, while natural compounds **1**, **6**, **8**, **10**, and **11** showed an uncompetitive type of inhibition (Table 1, Figs 5–8). Lineweaver-Burk plot was plotted to determine the inhibition type, secondary re-plot of Lineweaver-Burk plot was used to deduce (*K*i) values.

### Molecular docking studies

Sitemap analysis was run for non-competitive, uncompetitive, and mixed inhibitors to identify the potential allosteric binding site of the enzyme. Among the 5 top ranked sites, the druggable site with highest site score of 1.05 was chosen as allosteric site with respect to the thymidine for docking studies.

**Fig 9:**
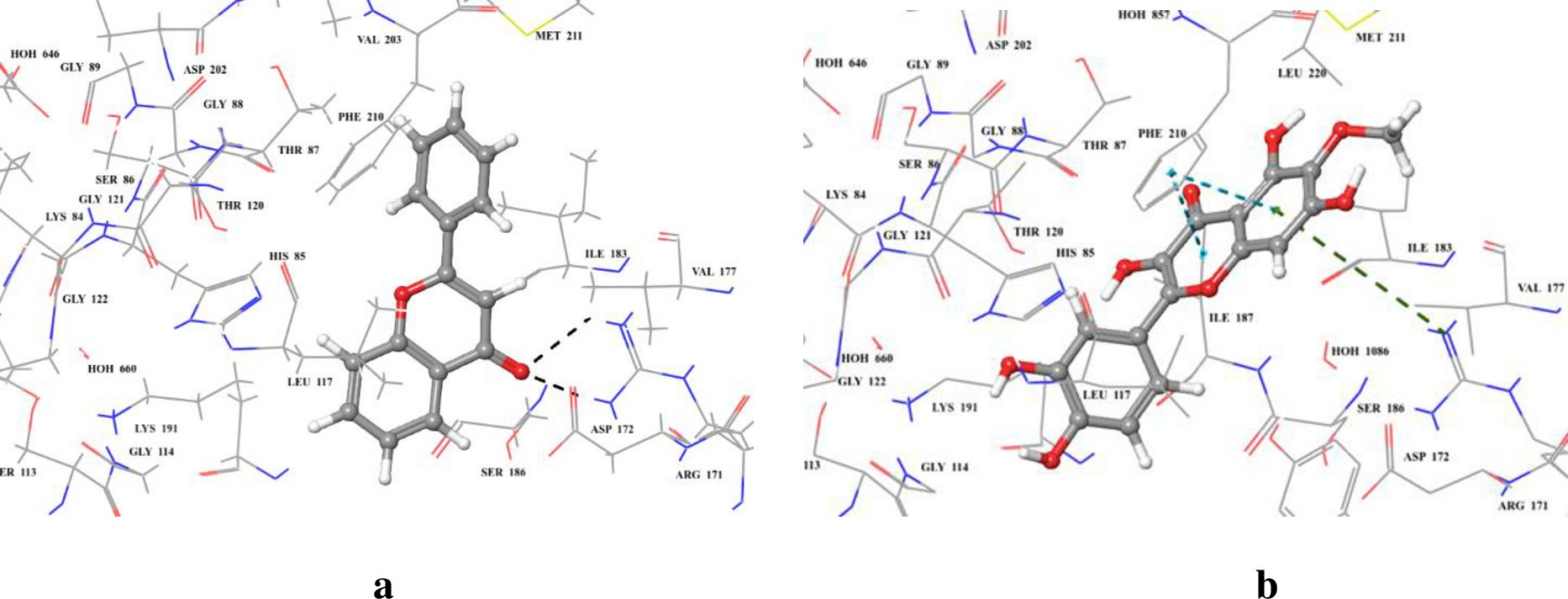
**3D modelled pose of (a) compound 1, and (b) compound 5 *via* docking** in the sitemap predicted binding site of *E*. *coli* TP. Hydrogen bonds are represented as black dotted lines. π-π and π-cation interactions are represented as blue and green dotted lines, respectively.

The docking analysis revealed that all of the *in silico* predicted lowest energy complexes were stabilized by stacking interaction, and intermolecular hydrogen bonds. Compound **1** having the primary flavonoid scaffold, form hydrogen bonds with Arg 171, while compound **5** with OH and OCH_3_ substitutions form π-cation interaction with Arg 171. π-π interactions were also observed with Phe 210 (Fig 9A and 9B).

MMGBSA score provides the predictive binding affinity of the ligand binding to the protein. A more negative score is indicative of better binding affinity towards corresponding ligand molecule. Analysis of MMGBSA score revealed that compounds **1**, and **5** have predictive binding energies of -40.45, and -61.99 kcal/mol, respectively, which is in agreement with their experimental *K*i values *i*.*e*., 225 and 150 μM, respectively.

**Fig 10:**
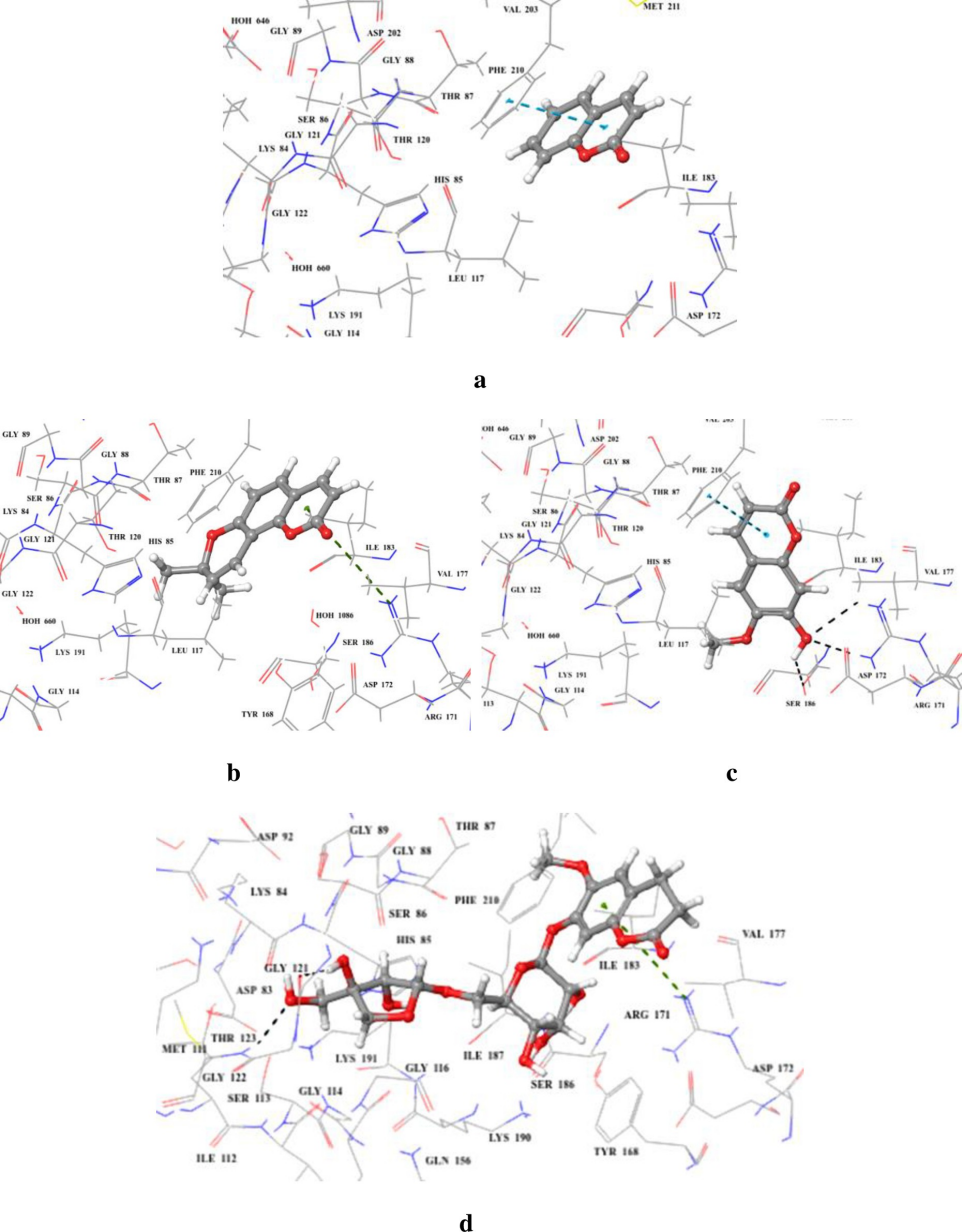
**3D modelled pose of (a) compound 6 (b) compound 8 (c) compound 9 and (d) compound 10 *via* docking** in the sitemap predicted binding site of *E*. *coli* TP. Hydrogen bonds are represented as black dotted lines. π-π and π-cation interactions are represented as blue and green dotted lines, respectively.

Molecular docking of coumarins with TP showed that compound **6** form only π-π interaction through its pyran ring with Phe210 (Fig 10A). The dimethyl pyran ring in compound **8** made π-cation interaction with Arg171 (Fig 10B). Presence of hydroxyl and methoxy groups in compound **9** form hydrogen bonds with Arg171, and Ser186, along with π-π interaction with Phe 210 (Fig 10C). Compound **10** showed π-cation interaction *via* its benzyl moiety to Arg171, while the furan ring made hydrogen bonds with Gly121, and Thr123 (Fig 10D). Correlation between the experimental *K*i values and predictive binding affinities (*via* MMGBSA scoring) were also observed in coumarins **6**, **8**, and **10**. Compound **10** with *Ki* value 145.0 ± 0.001 μM has exhibited the highest binding affinity with MMGBSA score of -91.01 kcal/mol. This can be attributed to the large size of the molecule that helped it to better accommodate into the allosteric site. Compounds **6** (*Ki* = 171 ± 0.001 μM), and **8** (*Ki* = 162.0 ± 0.001 μM) showed the predictive binding energies of -39.85, and -54.53 kcal/mol, respectively. Despite being substituted derivative of compound **6**, compound **9** did not show any correlation between experimental *K*i value and MMGBSA score relative to other derivatives of compound **6** *i*.*e*., compounds **8,** and **10**. The MMGBSA score for compound **6** was found to be -44.90 kcal/mol with *K*i value of 419.0 ± 0.001 μM.

**Fig 11:**
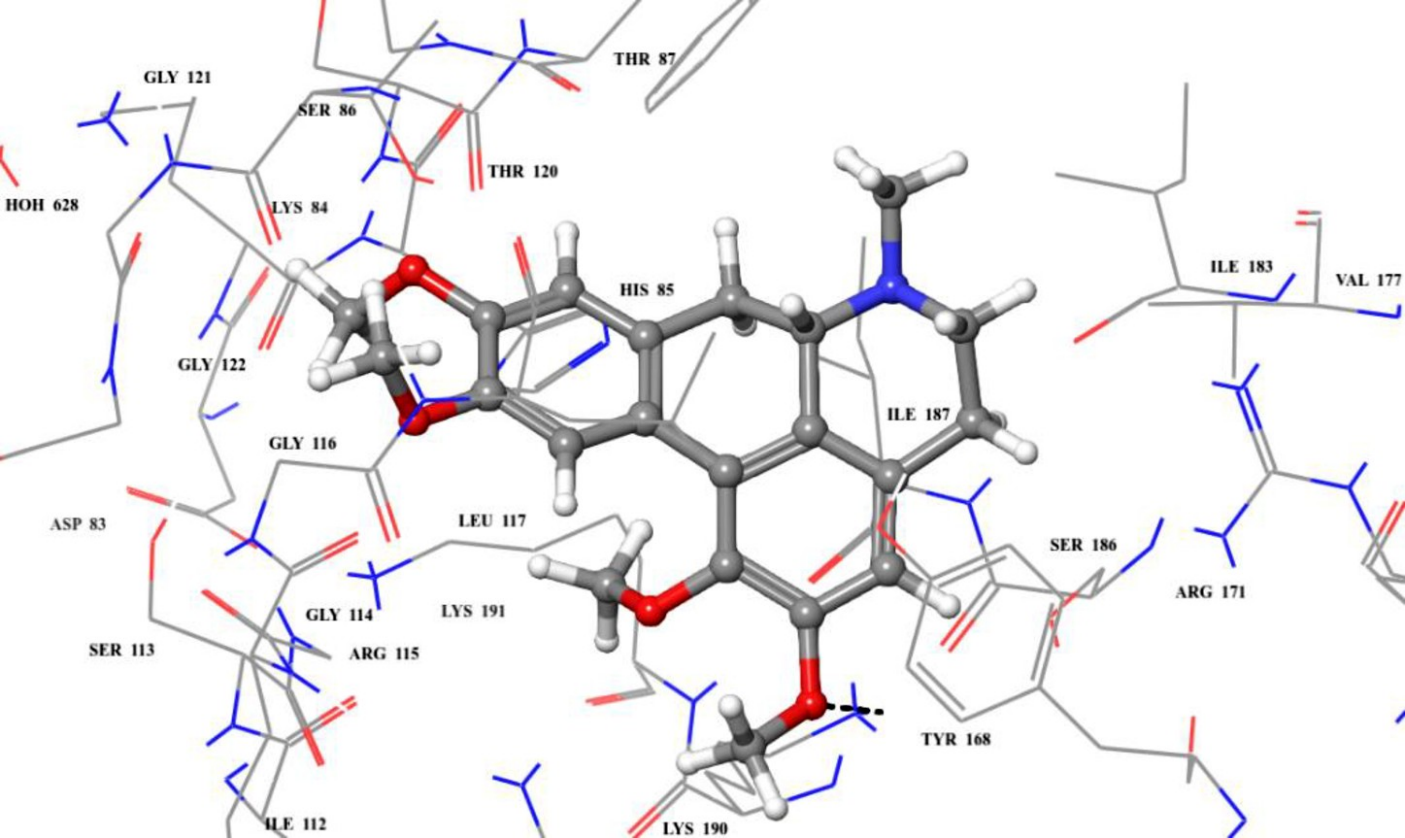
**3D modelled pose of (a) compound 11 *via* docking** in the sitemap predicted binding site of *E*. *coli* TP. Hydrogen bonds are represented as black dotted lines.

Molecular docking studies also showed that compound **11** had only one hydrogen bond with Lys 190 *via* one of its dimethoxy benzyl moieties (Fig 11). The predictive binding energy (ΔG_bind_) was found to be -41.79 kcal/mol.

**Fig 12:**
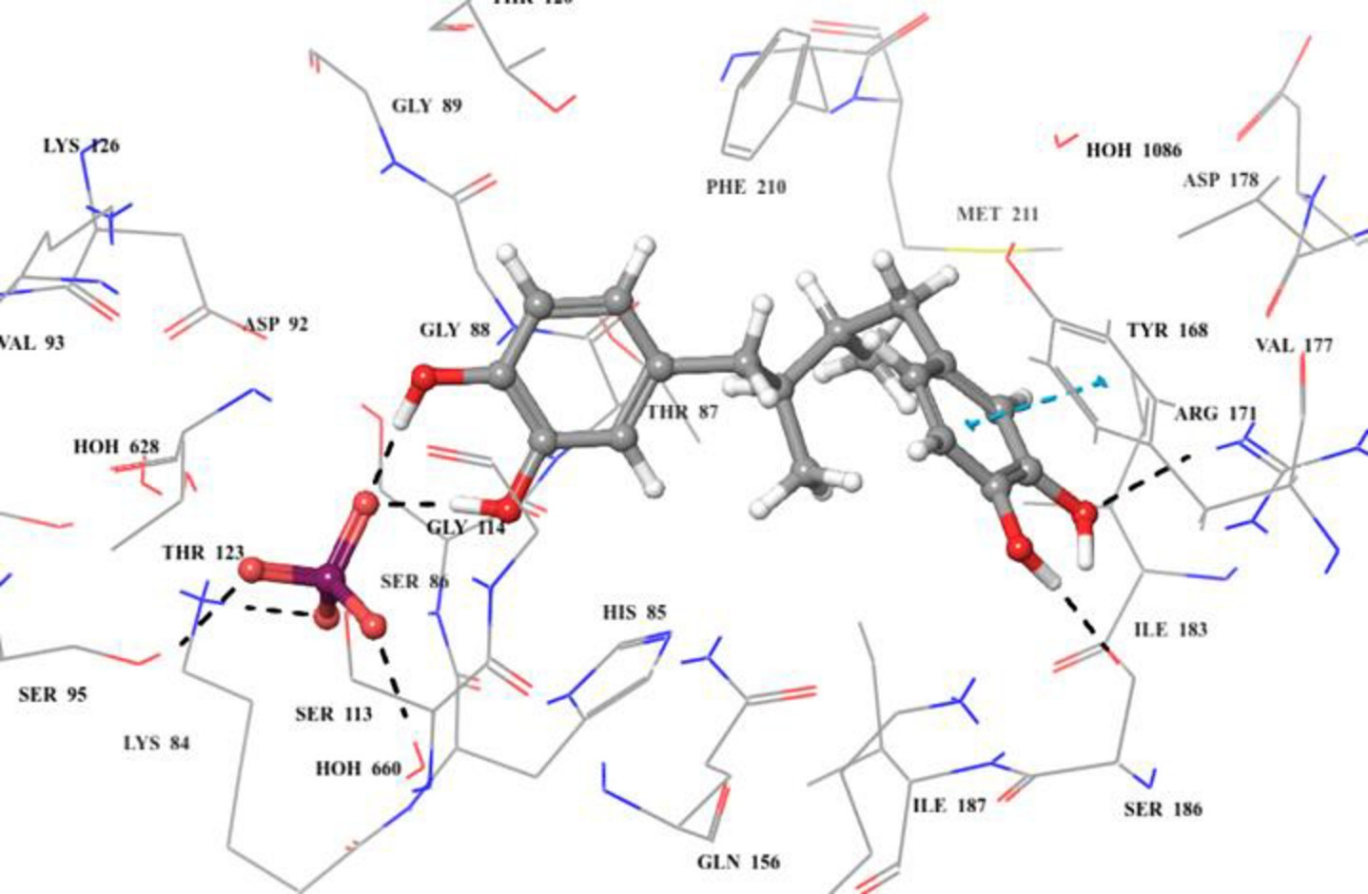
3D modelled pose of compound 18 *via* docking in the active site (thymidine binding site) of *E*. *coli* TP. Hydrogen bonds are represented as black dotted lines. π-π and π-cation interactions are represented as blue and green dotted lines, respectively.

Compound **18** of carboxylic acid class was found to be competing with thymidine for binding (competitive inhibitor); therefore it was docked into the substrate binding site of TP. Analysis of docking result showed that compound **18** adopted a different binding pose than substrate thymidine. One of the dihydroxy benzyl moieties interacted *via* π-cation interaction to Tyr 168. While the attached OH groups form hydrogen bonds with Arg 171, and Ile 183. The other dihyroxy benzyl group form hydrogen bonds with phosphate ion in substrate binding site (Fig 12). The compound was rescored *via* MMGBSA, and turned out to give predictive binding energy of about -60 kcal/mol.

### Cytotoxicity assay

Compounds **1**, **6**–**11**, **15**, and **18** were finally subjected to cytotoxicity MTT assay on 3T3 cell line. Compound **1** showed a weak growth inhibition of 3T3 cells (IC_50_ = 29.05 ± 0.61μM) in comparison to standard cycloheximide (IC_50_ = 0.20 ± 0.10 μM), while compounds **6**–**11**, **15**, and **18** were found to be non-cytotoxic. These compounds are, therefore, promising leads to be investigated further for *in vivo* anti- cancer and other relevant therapeutic activities.

## Conclusions

Many solid tumors have over-expression of thymidine phosphorylase (TP). TP assists cancer progression by inducing angiogenesis and preventing apoptosis. These critical functions make it an ideal target for the development of anti-angiogenic compounds. This systematic study identifies several natural products as anti-TP molecules with inhibitory activity good to weak, in comparison to the standard 7-deazaxanthine. Masoprocol (**18**), a dicatechol isolated from the *Larrea divaricata* Cav., showed the significant TP inhibitory activity, among all the natural products evaluated. It was found to interact with the active site residues of TP in kinetic studies (competitive inhibitor); however it exhibited a different binding pose than the natural substrate in *in silico* studies. Furthermore, it was non-cytotoxic to mouse fibroblast cells (3T3). These compounds can serve as leads for the development of new anti-angiogenic molecules.

## Supporting information

S1 Information Plant material used, extraction and isolation of natural compounds. (DOCX)

S1 Table Isolation and spectroscopic data of the natural compounds 1–18. (DOCX)
