# Supplementary material for: Natural compounds as angiogenic enzyme thymidine phosphorylase inhibitors: In vitro biochemical inhibition, mechanistic, and in silico modeling studies
Source: PLoS One. 2019 Nov 19;14(11):e0225056. doi: 10.1371/journal.pone.0225056 (PMC6863536; doi:10.1371/journal.pone.0225056)
Supplement: S1 Information — (DOCX) [file pone.0225056.s001.docx]

**Information S1: Plant material used, extraction and isolation of natural compounds**

**Materials:**

Solvents used for the extraction and purification were purchased from Sigma Aldrich, USA. Dichloromethane analytical standard; CAS Number: 75-09-2, hexanes analytical standard; CAS Number: 110-54-3, ethyl acetate analytical standard; CAS Number: 141-78-6, *n*-butanol analytical standard; CAS Number: 71-36-3, chloroform analytical standard; CAS Number: 67-66-3, petroleum ether ACS reagent; CAS Number: 8032-32-4, ethanol analytical standard; CAS Number: 64-17-5, acetone analytical standard; CAS Number: 67-64-1, methanol analytical standard; CAS Number: 67-56-1, Potassium hydroxide CAS Number: 1310-58-3. Sulfuric Acid CAS: 7664-93-9 was purchased from Thermo Fisher Scientific, USA. Silica gel (250–400/70-230 mesh; CAS No. 7631-86-9; E. Merck, Germany) was used for column chromatography; silica-gel 60 F254 plates CAT No. 105554E. Merck, Germany) were used for TLC. Sephadex LH-20 column (CAT Number LH20100) was purchased from Merck, Germany.

**Plant Materials for Compounds 1, 3, and 4**

The whole plant of *Potentilla evestita* L. was collected from Gilgit, Pakistan. It was identified by the Prof. Dr. Suraiya Khatoon, Taxonomist at Department of Botany, University of Karachi. Voucher specimen (No. 707) was deposited at the herbarium of Department of Botany, University of Karachi, Pakistan.

**Extraction and Isolation of Compounds 1, 3, and 4**

Shade dried whole plant (15.0 kg) of *P. evestita* was ground into fine powder, and soaked in 25 L ethanol for 10 days at room temperature. The resulting extract was filtered and evaporated under reduced pressure at 45°C to yield 300 g dark brown residue. The residue was suspended in water and subsequently extracted with solvents of increasing polarity, namely hexanes (3×10 L), ethyl acetate (EtOAc 3×12 L), and *n*-butanol (BuOH 1×3 L). Each extract evaporated under reduced pressure to obtain hexanes extract (70.0 g), EtOAc extract (80.0 g), and *n*-butanol (40.0 g).

The EtOAc soluble fraction was subjected to column chromatography on silica gel. The chromatographic separation with successive elution using mixtures of hexanes: EtOAc in increasing order of polarity gave six major fractions A (oil part; 30.0 g), B (2.0 g), C (0.8 g), D (0.7 g), and F (5.0 g). Fraction B was further purified by column chromatography eluting with hexanes: EtOAc (3:7) to afford compound **4** (12.0 mg). Fraction C was further purified by column chromatography eluting with hexanes: EtOAc (1.5:8.5) to afford compound **1** (10 mg), and with hexanes: EtOAc (3.5:6.5) to obtain compound **3** (10.3 mg).

**Plant Material for Compound 2**

The whole plant material of *Eremostachys loasifolia* Benth. was collected from Miuan Ghundi near Lakpass, Quetta Valley, Balochistan, Pakistan, and taxonomically identified by Prof. Dr. Rasool Bakhsh Tareen, Taxonomist at Department of Botany, University of Balochistan, where a voucher specimen (No. el. Rbt. 01.2005) was deposited in the herbarium.

**Extraction and Isolation of Compound 2**

The air-dried whole plant material *E. loasifolia* (20.0 kg) was extracted with EtOH (3×50 liters each 10 days) at room temperature. The solvent was evaporated to yield the residue (750 g), which was divided into hexanes (81.0 g), chloroform (CHCl_3_: 70.0 g), EtOAc (200 g), *n*-BuOH (163.5 g), and water-soluble (63.0 g) sub-fractions. The CHCl_3_-soluble fraction was subjected to column chromatography over silica gel eluting with hexanes–CHCl_3_, CHCl_3_, CHCl_3_–MeOH in increasing order of polarity to obtain three major fractions A–C. Fraction B obtained from CHCl_3_–MeOH (9.8:0.2) was further purified by column chromatography eluting with CHCl_3_–MeOH (9.5:0.5) to obtain compound **2** (8.0 mg).

**Plant Material for Compound 5**

Flowers of *Tegetes patula* Linn. were collected during 2000 and 2003 from Karachi University Campus, identified by Dr. Rubina Dawar of the Department of Botany, University of Karachi, and a voucher specimen (No. 67280) was deposited in the herbarium.

**Extraction and Isolation of Compound 5**

The fresh, uncrushed, orange red flowers of *T. patula* (181.0 g) were extracted twice with methanol (MeOH). MeOH extract was then concentrated in vacuo and kept overnight at room temperature, affording a brownish insoluble matter that turned black on exposure to light and air. This was separated by filtration. The filtrate on evaporation of the solvent furnished a residue (17.0 g) that was partitioned between distilled water and petroleum ether (PE). The aqueous phase was extracted successively with CHCl_3_ (three times), EtOAc (six times) and BuOH (three times). Each phase (PE, CHCl_3_, EtOAc, and BuOH) was washed with water, dried over anhydrous sodium sulfate and evaporated under reduced pressure that provided respective residues. The first three EtOAc phases showed a single spot on TLC, they were thus pooled to obtain single fraction EA1 (1.2 g) which was identified as compound **5** (31.0 mg) through spectral studies.

**Plant Material for Compound 6**

The whole plant material of *Eremostachys vicaryi* Benth. was collected from Ziarat Valley of Balochistan Province of Pakistan, and identified by Prof. Dr. Rasool Bakhsh Tareen, Plant Taxonomist, Department of Botany, University of Balochistan, Quetta, where a voucher specimen (No. el. Rbt. 02. 2005) was deposited in the herbarium.

**Extraction and Isolation of Compound 6**

The shade-dried whole plant material *E. vicaryi* Benth (20.0 kg) was extracted with EtOH (3×40 liters, 10 days each) at room temperature. The extract was obtained by evaporating solvent under reduced pressure to yield a residue (500 g), which was partitioned into hexanes (130 g), CHCl_3_ (100 g), EtOAc (60.0 g), *n*-BuOH (80.0 g), and water-soluble (100 g) sub-fractions. The CHCl_3_-soluble fraction was subjected to column chromatography over silica gel eluting with hexanes–CHCl_3_, CHCl_3_, CHCl_3_–MeOH with gradient elution to yield five major fractions A–E. Fraction A obtained from hexanes: CHCl_3_ (6.0:4.0) was re-chromatographed on silica gel, and eluted with hexane:CHCl_3_ (6.0:4.0) to obtain compound **6** (21.0 mg).

**Plant Material for Compounds 7, and 8**

The seeds of Indian celery (*Seseli diffusum* (Roxb. ex Sm)) were provided and identified by Prof. Jean-Pierre Reduron, (Directeur de la Mission Developpement Durable, Responsable du Conservatoire Botanique, Communaute de l’Agglomeration Mulhouse Sud Alsace, France) and by Prof. Dr. Michel Farille, from Herbarium FARILLE, Jardin Botanique Alpin La Jaysinia, Samoens, Hte-Savoie, France. The voucher specimen number n 84-272 was documented in the same section.

**Extraction and Isolation of Compounds 7, and 8**

Indian celery seeds (100 g) were repeatedly extracted with hexane, CHCl_3_, acetone and MeOH (4×400 mL, 4 h under reflux for each solvent). This yielded hexanes (1.1 g), CHCl_3_ (2.2 g), acetone (5.0 g), and MeOH (18.0 g) viscous extracts. The silica gel column chromatography of the hexane extract was carried out with hexanes–EtOAc and EtOAc–MeOH gradient elution to afford five fractions (A-E). Fraction A obtained from hexane was re-chromatographed on silica gel by using hexanes–EtOAc (1.0:3.0) to obtain compounds **7** (14.0 mg), and **8** (43.0 mg).

**Plant Material for Compounds 9, and 10**

The stem bark of *Pauridiantha callicarpoides* (Hiern) Bremek. was collected from the Dja rain forest in the eastern region of Cameroon in December of 2010. The plant was taxonomically authenticated by Mr. Victor Nana, botanist at the National Herbarium of Cameroon in Yaoundé, where a voucher specimen (No. 39807=SPDK) was deposited.

**Extraction and Isolation of Compounds 9, and 10**

The air-dried stem bark (2.8 kg) was powdered and extracted with CH_2_Cl_2_-MeOH (1: 1, 10 L) twice at room temperature for 48 h and 12 h, respectively. The solvent was evaporated under reduced pressure to afford a crude extract (345.4 g) which was subjected to silica gel column chromatography with CH_2_Cl_2_-MeOH solvent system of increased polarity to give 5 fractions: A (CH_2_Cl_2_, 9.3 g); B [CH_2_Cl_2_-MeOH (2.5 – 12.5%), 17.97 g]; C [CH_2_Cl_2_-MeOH (12.5 – 22.5%), 52.2 g]; D [CH_2_Cl_2_-MeOH (22.5 – 30%), 50.92 g], and E [CH_2_Cl_2_-MeOH (30 – 50%), 45.5 g]. Repeated silica gel column chromatography of fraction A eluted with a gradient of hexanes-EtOAc yielded compound **9** (2.0 g). Compound **10** (1.8 g) was obtained after further purification of fraction C over a silica gel column, eluting with CH_2_Cl_2_-MeOH (1.5 – 30%).

**Plant Material for Compound 11**

The plant material *Glaucium flavum* Cr. was collected on November 2010 from Sidi Barrani, at the north coast of Egypt. The plant was identified by Prof. Dr. Samih I. Eldahmy, Professor of Pharmacognosy, Faculty of Pharmacy, Zagazig University. Voucher specimen (number P145) was deposited in the herbarium of the Department of Pharmacognosy, Zagazig University, Egypt.

**Extraction and Isolation of Compound 11**

The air-dried powdered aerial parts (1.1 kg) were repeatedly extracted with ethanol 70% (5×3 L) at room temperature. The solvent was evaporated under reduced pressure to afford ethanol extract residue (182 g). The residue was fractionated through an acid–base extraction. The residue was suspended in a 5% aqueous solution of HCl (1 L) and extracted with methylene chloride (0.5 L × 6 extractions) to yield the acidic methylene chloride fraction (29.30 g). This acidic fraction was dissolved in chloroform: methanol (2:1), and crystallization was enabled yielded compound **11** (1.17 g).

**Plant Material for Compound 12**

*Berberis jaeschkeana* Schneid var. *jaeschkeana* stem was collected from Azad Kashmir Pakistan during July 2009 and was identified by Prof. Dr. Tanveer Akhtar (Chairperson Botany Department, University of Azad Jammu and Kashmir). A voucher specimen bearing number 9615-B was deposited in the herbarium of the Botany Department University of Peshawar.

**Extraction and Isolation of Compound 12**

Stem wood with bark (5.0 kg) was first shade dried at room temperature and then pulverised with heavy duty grinder. The powdered plant material was soaked in methanol at room temperature for 10 days. The solvent was evaporated under reduced pressure to yield a dark brownish black residue (226 g). The residue was then treated with 5% aqueous HCl solution, filtered and allowed to stand overnight to afford fraction A (76.0 g). The residue was then repeatedly extracted with dichloromethane (800 mL × 4), using separating funnel to afford fraction B (13.0 g). After this the residue was basified with ammonia (NH_4_OH) to pH 9 and then extracted with EtOAc to afford Fraction C (32.0 g). Fraction A was then subjected to silica gel column chromatography and the column was eluted with chloroform: methanol with increasing the polarity gradually to obtain compound **12** (307.0 mg).

**Plant Material for Compound 13**

*Loranthus micranthus* Linn*.* leaves, were collected in April 2007 from different locations in Nsukka LGA, Enugu State. The leaves were identified and certified by Mr. A. O. Ozioko, a Taxonomist of the Bioresources Development and Conservation Programme (BDCP), Nsukka, Enugu State. Voucher specimen bearing number BDCP-532-07 was kept at the BDCP Center.

**Extraction and isolation of Compound 13**

Shade dried leaves of *L. micranthus* were pulverized to produce fine powder (1 kg). It was then extracted in batches with 6.5 L of 98% aqueous methanol using a Soxhlet extractor. The solvent was evaporated *in vacuo*, at 45 ± 5°C to yield viscous extract (210.5 g). Later, 50 g of the extract was adsorbed on silica Gel (mesh size 60), eluted repeatedly with hexane, chloroform, acetone, ethyl acetate, methanol, and water to collect the respective solvent fractions, until all the constituents retrieved by a particular solvent, separately.

Chloroform soluble fraction (6.0 g) of was separated on silica gel with isocratic system of chloroform: methanol (19:1) to afford three fractions F1-F3. The column was continued to wash further with chloroform: methanol (9:1), (8:1), and (7:1) to yield F4, F5, and F6 fractions, respectively. The F1 (1.8 g) was further sub-fractionated on silica gel with the gradient elution of chloroform, chloroform: methanol (49:1; 39:1; 29:1; 19:1; 9:1) and methanol (100%), and aliquots of 25 mL were collected to obtain three major fractions E1-E3. An alkaloidal compound **13** (150.0 mg) was purified as white brittle crystals from E2 sub-fraction while evaporating the solvent after phase separation.

**Plant Material for Compound 14**

The plant *Delphinium nordhagenii* Wendelbo was collected from the village Kalam, Swat District, N. W. F. P. Pakistan, in August 2003. Botanical identification was done by Dr. Habib Ahmad, Jehanzeb Post Graduate College, Saidu Sharif, Swat. A voucher specimen (ND-09) was deposited in the herbarium of the Botany Department.

**Extraction and Isolation** **of Compound 14**

Dried and powdered aerial parts (4 kg) of the plant were extracted exhaustively with *n*-hexane (3 × 8 L) followed by 80% EtOH (3 × 10 L) at room temperature for 7 days (3 times) The filtrate was evaporated in vacuo to yield 460 g of residue. The residue was acidified to pH 2 by 5% H_2_SO_4_ and extracted with CH_2_Cl_2_ (3 × 2 L) to obtain a mixture (39.5 g). The acidic aqueous solution was basified (pH, 8-10) by using 10% KOH and extracted with CH_2_Cl_2_ (5 × 2 L) to yield 6.5 g of crude mixed alkaloids. The crude alkaloidal mixture (2.55 g) obtained was fractionated on a silica gel column, and five combined fractions were obtained. On repeated flash column chromatography using hexane-acetone-Et_2_NH (9:1:10 drops per 100 mL) compound **14** (5.0 mg) was purified.

**Plant Material for Compound 15**

The whole plant of *Spiraea* *brahuica* Boiss. (10 kg) was collected from Ziarat Valley, Balochistan region and identified by plant taxonomist Prof. Dr. Rasool Bakhsh Tareen, Department of Botany, University of Balochistan, Pakistan where a voucher specimen (No. SB.R.B.T.08.BUH) has been deposited in its Herbarium.

**Extraction and Isolation of Compound 15**

The plant material (10 kg) was shade-dried, ground, and extracted with ethanol (3×40 L). The combined ethanolic extract was evaporated in vacuum to obtain a crude residue (450 g), which was divided into fractions soluble in *n*-hexane (70.0 g), CHCl_3_ (40.0 g), EtOAc (12.0 g), *n*-BuOH (250 g), and H_2_O (40.0 g) by liquid-liquid extraction. The CHCl_3_ soluble fraction was subjected to column chromatography eluting with mixtures of hexanes–EtOAc gradiently to furnish three major fractions A–C. Fraction A eluted with hexanes–EtOAc (7:3) was further chromatographed on silica gel column and eluted with hexanes–EtOAc (8:2) to obtain two successive fractions A1 and A2. Compound **15** (15.0 mg) was purified from fraction A1 by using hexanes : EtOAc (4:6) solvent system.

**Plant Material for Compound 16**

The aerial parts of *Ipomoea carnea* Jacq. ssp. *fistulosa* were collected from Karachi, Pakistan, in July 2010. It was identified by Taxonomist Dr. Sher Wali Khan, a voucher specimen (No-85855) was deposited at the Department of Botany, University of Karachi.

**Extraction and Isolation of Compound 16**

Air-dried aerial parts of plant (20 kg) were percolated three times (each 3 days) with 80 % methanol-water at room temperature. The resulting methanolic extract was concentrated under reduced pressures to obtain a crude methanolic extract (120 g), which was then partitioned into petroleum ether (14.1 g), CHCl_3_ (26.0 g), EtOAc (19.0 g), BuOH (38.6 g), and water layers (27.2 g). The EtOAc extract was subjected to column chromatography (silica gel) by using EtOAc: hexanes (10-100 %) gradient solvent system to obtain 5 fractions (A-E). Fraction B (225 mg) was further subjected to silica gel column chromatography by using DCM: hexanes (20-100 %) as eluent to obtain three sub fractions (F1-F3). The sub-fractions F-2 and F-3 were combined together and subjected to column chromatography by using acetone: hexanes (50 %) as mobile phase to obtain compound **16** (10.8 mg).

**Plant Material for Compound 17**

The aerial parts of *Bistorta manshuriensis* Komarov were collected from Mt. Daeduk, Gangwon Province, Korea, in June 2008. A voucher specimen (SKKU-2008-6) of the plant was deposited in the herbarium of the School of Pharmacy, Sungkyunkwan University, Suwon, Korea.

**Extraction and Isolation of Compound 17**

The aerial parts of the plant (2.9 kg) were extracted with 80% MeOH at room temperature three times, after which solvent was evaporated under reduced pressure to give a viscous MeOH extract (219 g), which was dissolved in water (800 mL) and then successively partitioned with hexane (10.0 g), CH_2_Cl_2_ (2.0 g), EtOAc (9.0 g), and *n*-BuOH (32.0 g) soluble fractions. The EtOAc fraction was separated by successive chromatographic methods (silica gel column, RP-C18 silica gel column, Sephadex LH-20 column and RP-C18 prep. HPLC) to obtain compound **17** (4.0 mg).

**Plant Material for Compound 18**

Aerial parts of *Larrea nitida* Cav. were collected in December 2008, on Bauchaceta locality, Iglesia district, province of San Juan (Argentina). The plant was identified by Dra. Gloria Barboza, IMBIV (Instituto Multidisciplinario de Biología Vegetal), Facultad de Ciencias Exactas, Físicas y Naturales, Universidad Nacional de Córdoba, Argentina. A voucher specimen (CORD 1335) was documented at the herbarium of the Botanic Museum of Córdoba.

Raw propolis samples were kindly provided by beekeepers from the Bauchaceta district. Hives were located in the same geographical area where *L. nitida* grows between 2500 and 2700 m.a.s.l. at pre-mountain area close to the Andes range. Samples of propolis were collected during 2008, using propolis traps. Reference propolis samples were deposited at the Instituto de Biotecnología, Universidad Nacional de San Juan, identified as LnP1, LnP2 and LnP3.

**Extraction and Isolation of Compound 18**

One hundred grams of propolis associated with plant *Larrea nitida* Cav. were successively extracted at room temperature (3×24 h) with petroleum ether (PE), dichloromethane (DCM), EtOAc, and EtOH. Then, solvents were evaporated under vacuum to give semisolid extracts of petroleum ether (12.6 %), dichloromethane (37.6 %), ethyl acetate (37.2 %), and ethanol extract (5 %). The ethyl acetate extract (5.2 g) was loaded on a Sephadex LH-20 column (eluant: MeOH). After pooling, 15 fractions (A1-A15) were obtained. Separation of A5 (294 mg) by preparative reverse phase HPLC with MeOH: H_2_O (70:30) as mobile phase afforded **18** (25 mg). The fraction A8 (410 mg) was successively subjected through a Sephadex LH-20 column (eluant: MeOH) and by preparative HPLC (eluant: MeOH: H_2_O, 70:30) to yield 99.2 mg of compound **18**.
