# Supplementary material for: Natural compounds as angiogenic enzyme thymidine phosphorylase inhibitors: In vitro biochemical inhibition, mechanistic, and in silico modeling studies
Source: PLoS One. 2019 Nov 19;14(11):e0225056. doi: 10.1371/journal.pone.0225056 (PMC6863536; doi:10.1371/journal.pone.0225056)
Supplement: S1 Table — (DOCX) [file pone.0225056.s002.docx]

| **Table S1 : Isolation and spectroscopic data of the natural compounds 1-18.** | | | | | |
| --- | --- | --- | --- | --- | --- |
| **Compound** | **Plant/ Part of Plant** | **Fraction** | **Yield** | **Spectroscopic Data** | **Reference** |
|   **1** | *Potentilla evestita* L. /Whole plant (Methanolic extract) | Ethyl acetate | 10.0 mg | ^1^H NMR (DMSO-*d*_6_, 400 MHz) δ 6.96 (1H, s, H-3), 8.02 (1H, m, H-5), 7.43 (1H, ddd, *J* = 1.1, 7.0, 7.9 Hz, H-6),7.76 (1H, ddd, *J* = 1.6, 7.0, 8.3 Hz, H-7), 7.67 (1H, dd, *J* = 1.1, 8.3 Hz, H-8), 8.01 (1H, m, H-2ʹ), 7.51 (1H, m, H-3ʹ), 7.53 (1H, m, H-4ʹ), 7.53 (1H, m, H-5ʹ), 8.01 (1H, m, H-6ʹ). | 58 |
|   **2** | *Eremostachys loasifolia* Benth. /Whole plant (Ethanolic extract) | Chloroform soluble fraction | 12.5 mg | ^1^H NMR (CDCl_3_, 400 MHz) δ 6.78 (1H, s, H-3), 6.36 (1H, d, *J*=1.9 Hz, H-6), 6.47 (1H, d, *J*=1.9 Hz, H-8), 7.76 (1H, d, *J*=8.4 Hz, H-4ʹ), 7.03 (1H, d, *J*=8.4 Hz, H-5ʹ), 3.87 (3H, s, OCH_3_-7), 3.98 (3H, s, OCH_3_-6ʹ), 11.69 (OH-5). | 38 |
|   **3** | *Potentilla evestita* L. /Whole plant (Methanolic extract) | Ethyl acetate | 10.3 mg | ^1^H NMR (DMSO-*d*_6_, 200 MHz) δ 6.81 (1H, s, H-2), 6.21 (1H, d, *J*= 2.1 Hz, H-6), 6.50 (1H, d, J= 2.1 Hz, H-8), 7.95 (2H, d, *J*= 8.8 Hz, H-2ʹ, H-6ʹ), 6.94 (2H, d, *J*= 8.8 Hz, H-3ʹ, H-5ʹ), 10.39 (1H, s, OH-4ʹ), 12.94 (1H, s, OH-5), 10.80 (1H, s, OH-7). | 59 |
|   **4** | *Potentilla evestita* L. /Whole plant (Methanolic extract) | Ethyl acetate | 12.0 mg | ^1^H NMR (Acetone-*d*_6_, 500 MHz) δ 6.28 (1H, d, *J*= 1.9 Hz, H-6), 6.57 (1H, d, *J*= 1.9 Hz, H-8), 6.76 (1H, s, H-3), 7.58 (2H, m, H-3ʹ, H-5ʹ), 7.58 (1H, m, H-4ʹ), 8.05 (2H, dd, *J* = 8.1, 1.8 Hz, H-2ʹ, H-6ʹ), 12.88 (1H, sl,OH-5). | 60, 61 |
|   **5** | *Tegetes patula* Linn. /Flower (Methanolic extract) | Ethylacetate | 31.0 mg | ^1^H NMR (Acetone-*d*_6_, 300 MHz) δ 6.59 (1H, s, H-8), 7.82 (1H, d, *J*= 2.2 Hz, H-2ʹ), 6.98 (1H, d, *J*= 8.4 Hz, H-5ʹ), 7.69 (1H, dd, *J*= 8.4, 2.1 Hz, H-6ʹ), 9.45 (br.s, 3-OH ^a,b^), 12.20 (s, 5-OH ^a^), 9.28 (s, 3ʹ-OH ^a,b^), 9.28 (s, 4ʹ-OH ^a,b^), 3.87 (3H, s, 6-OCH_3_).  ^a^ Disappeared with D_2_O shake ^b^ Assignment may be interchanged | 40,  62 |
| ****  **6** | *Eremostachys vicaryi* Benth. /Whole plant (Ethanolic extract) | Chloroform soluble fraction | 21.0 mg | ^1^H NMR (CDCl_3_, 400 MHz) δ 6.37 (1H, d, *J* = 9.5 Hz, H-3), 7.66 (1H, d, *J*= 9.6 Hz, H-4), 7.51 (1H, d, *J*= 9.6 Hz, H-5), 7.21 (1H, t, *J* = 9.5 Hz, H-6), 6.37 (1H, d, *J* = 8.3 Hz, H-8), 7.40 (1H, d, *J* = 7.3 Hz, H-7). | 63,64 |
|   **7** | *Seseli diffusum* (Roxb. ex Sm.) Sant. & Wagh. /Seeds of plant  (Methanolic extract) | Hexane | 14.0 mg | ^1^H NMR (CDCl_3_, 400 MHz) δ 4.27 (3H, s, OCH_3_), 6.26 (1H, d, *J*= 9.8 Hz, H-3), 7.02 (1H, d, *J*= 2.5 Hz, H-3ʹ), 7.12 (1H, s, H-8), 7.59 (1H, d, *J* = 2.5 Hz, H-2ʹ), 8.15 (1H, d, *J* = 9.8 Hz, H-4). | 42, 65 |
|   **8** | *Seseli diffusum* (Roxb. ex Sm.) Sant. & Wagh /Seeds of plant  (Methanolic extract) | Hexane | 43.0 mg | ^1^H NMR (CDCl_3_, 100 MHz) δ 1.46 (6H, s), 5.67 (1H, d, *J*= 10.0 Hz), 6.17 (1H, d, *J*= 9.0 Hz), 6.66 (1H, d, *J*= 8.0 Hz), 6.85 (1H, d, *J*= 10.0 Hz), 7.18 (1H, d, *J*= 8.0 Hz), 7.57 (1H, d, *J*= 9.0 Hz). | 42,  66 |
|   **9** | *Pauridiantha callicarpoides* (Hiern) Bremek.*/*Stem bark (Dicholromethane/methanol) | Dicholromethane | 2.0 g | ^1^H NMR (CD_3_OD, 400 MHz) δ 6.23 (1H, d, *J*= 9.2 Hz, H-3), 7.88 (1H, d, *J*= 9.6 Hz, H-4), 7.14 (1H, s, H-5), 6.79 (1H, s, H-8), 3.93 (3H, s, OCH­­­_3_). | 67,  68,  43 |
|   **10** | *Pauridiantha callicarpoides*(Hiern) Bremek. */*Stem bark (Dicholromethane/methanol) | Dicholromethane/methanol | 1.8 g | ^1^H NMR (DMSO-*d*_6_, 90 MHz) δ 7.73 (d, *J*= 9.5 Hz, H-4), 6.13 (d, *J*= 9.5 Hz, H-3), 7.08 (1H, s, H-5), 6.95 (1H, s, H-8), 5.12 (1H, d, *J*= 4.7 Hz, H-1ʹ), 4.9 (1H, s, *J*= 3.0 Hz, H-1ʹʹ), 4.60, 4.62, 4.68, 4.74 (4H, each singlet, sugar hydroxyls), 4.42 (1H, t, 5ʹʹ-OH), 3.78 (1H, s, 3ʹʹ-OH), 3.85 (3H, s, OMe), 3.1-3.6 (9H, m, other sugar proton). | 43,  69 |
|   **11** | *Glaucium flavum* Cr. /Arial parts of plant (70% Ethanolic extract) | Acidic methylene chloride | 1.17 g | ^1^H NMR (DMSO-*d*_6_, 600 MHz) δ 6.88 (1H, s, H-3), 2.98 (2H, dd, *J*= 17.4 & 3.0 Hz, H-4), 3.40 (2H, dd, *J*= 13.5 & 3.6 Hz, H-5), 2.70 (1H, t, *J*=13.8 Hz, H-6a), 3.13-3.19 (2H, m, H-7), 6.99 (1H, s, H-8), 7.87 (1H, s, H-11), 3.62 (3H, s, H-1ʹ), 3.80 (3H, s, H-2ʹ), 3.07 (3H, s, H-6ʹ), 3.83 (3H, s, H-9ʹ), 3.77 (3H, s, H-10ʹ). | 70, 71 |
|   **12** | *Berberis jaeschkeana* Schneid var. *jaeschkeana* /Stem (Methanolic extract) | HCl | 307 mg | ^1^H NMR (CD_3_OD, 600 MHz) δ 3.25 (2H, t, *J*= 6.3 Hz, H-5), 4.10 (3H, s, H-10 (OCH­_3_), 4.20 (3H, s, H-9 (OCH_3_), 4.93 (2H, t, *J*= 6.3 Hz, H-6), 6.10 (2H, s, H-2), 6.95 (1H, s, H-4), 7.64 (1H, s, H-14), 7.99 (1H, d, *J*= 9.1 Hz, H-12), 8.10 (1H, d, *J*= 9.1 Hz, H-11), 8.69 (1H, s, H-13), 9.76 (1H, s, H-8). | 72, 73, 74 |
|   **13** | *Loranthus micranthus* Linn /Leaves (Mthanolic extrat) | Chloroform | 150 mg | ^1^H NMR (C_6_D_6_, 600 MHz) δ 1.24 (1H, br. s, H-1), 1.79 (1H, d mult, *J^2^*=13.0 Hz, H-2ɑ), 1.36 (1H, t mult^b^ *J^2^*=14.0^b^ Hz, H-2β), 2.35 (1H, tdt, *J^2^*=14.5 Hz, H-3ɑ), 1.36 (1H, d mult^b^ , *J^2^*=15.0 Hz, H-3β), 2.58 (1H, ddt, *J^2^*=11.2 Hz, H-4ɑ), 1.76 (1H,ddd, *J^2^*=11.2 Hz, H-4β), 2.52 (1H, dddd, *J^2^*=11.4 Hz, H-6ɑ), 1.57 (1H, td, *J^2^*=12.0 Hz, H-6β), 1.42 (1H, qt, *J^2^*=12.8 Hz, H-7ɑ), 1.34 (1H, d sext, *J^2^*=13.0 Hz, H-7β), 1.58 (1H, d mult, *J^2^*=13.0, H-8ɑ), 1.05 (1H, qt, *J^2^*=12.9 Hz, H-8β), 1.73 (1H, tdd, *J^2^*=12.8 Hz, H-9ɑ), 1.26 (1H, d quin, *J^2^*=12.8 Hz, H-9β), 1.80 (1H, dt, H-10), 3.80 (1H, ddd, *J^2^*=10.8 Hz, H-11-pro-*R*, 4.22 (1H, ddd, *J^2^*=10.8 Hz, H-11-pro-*S*).  ^b^ Determined from the 2D δ_C_/δ_H_ correlation spectrum. | 75, 76, 77 |
|   **14** | *Delphinium nordhagenii* Wendelbo */*Arial parts  (*n*-Hexane) | Dichloromethane | 5 mg | ^1^H NMR (CDCl_3_, 600 MHz) δ 0.81 (3H, s, C-18), 1.04 (3H, t, *J*= 7.2 Hz, *N*-CH_2_CH_3_), 3.22 (3H, s, OCH_3_), 3.31 (3H, s, OCH_3_), 3.41 (3H, s, OCH_3_), 3.70 (1H, t, *J*= 4.59 Hz, C-14), 4.85 and 4.98 (1H, each s, CH_2_). | 44 |
|   **15** | *Spiraea brahuica* Boiss /Whole plant (Ethanolic extract) | Chloroform | 15 mg | ^1^H NMR (CDCl_3_, 300 MHz) δ 7.36-7.65 (5H, m, aromatic H), 7.82 (1H, d, *J*= 15.9 Hz, H-3), 6.48 (1H, d, *J*= 15.9 Hz, H-2). | 78, 79 |
|   **16** | *Ipomoea carnea* Jacq. ssp. Fistulosa/ Arieal parts of plant (Methanolic acid) | Ethyl acetate | 10.8 mg | ^1^H NMR (Acetone-*d_6_*, 400 MHz) δ 7.16 (2H, s, H-3, H-7). | 80, 81 |
|   **17** | *Bistorta manshuriensis* Komarov /Arieal parts of plant (80 % Methanolic extract) | Dichloromethane | 4.0 mg | ^1^H NMR (CD_3_OD, 500 MHz) δ 7.58 (d, *J* = 2.0 Hz, H-2), 7.54 (1H, dd, *J*= 8.5, 2.0 Hz, H-6), 6.82 (1H, d, *J*= 8.5 Hz, H-5), 3.89 (3H, s, OCH_3_). | 82 |
|   **18** | *Larrea nitida* Cav. /Resins and plant exudates | Ethyl acetate | 99.2 mg | ^1^H NMR (CD_3_OD, 250 MHz) δ 0.79 (d, *J* = 6.0 Hz, 6H, 2-CH_3_, 3-CH_3_), 1.66 (q, *J* = 7.0 Hz, 2H, H-2, H-3), 2.12 (dd, *J* = 13.0, 9.0 Hz, 2H, H-1a, H-4a), 2.61 (dd, *J* = 13.0, 6.0 Hz, 2H, H-1b, H-4b), 4.98 (br. s, 4H, OH), 6.37 (dd, 2H, *J* = 7.8, 1.2 Hz, H-6ʹ, H-6ʹʹ), 6.62 (d, 2H, *J* = 1.2 Hz, H-2ʹ, H-2ʹʹ), 6.73 (d, 2H, *J* = 7.8 Hz, H-5ʹ, H-5ʹʹ). | 83,  84, 85, 86 |

**References :**

58 Moon BH, Lee YS, Shin CS, Lim YH. Complete assignments of the ^1^H and ^13^C NMR data of flavone derivatives. Bull Korean Chem Soc. 2005; 26: 603-608.

59 Fathiazad F, Delazar A, Amiri R, Sarker SD. Extraction of flavonoids and quantification of rutin from waste tobacco leaves. Iran J Pharm Sci. 2010; 5(3): 222-227.

60 Mouffok S, Haba H, Lavaud C, Long C, Benkhaled M. Chemical constituents of *Centaurea omphalotricha* Coss. & Durieu ex Batt. & Trab. Rec Nat Prod. 2012; 6(3), 292-295.

61 Antri A, Messouri I, Tlemçani R, Bouktaib M, El Alami R, El Bali B, Lachkar M. Flavone glycosides from *Calycotome villosa* Subsp. Intermedia. Molecules. 2004; 9(7) : 568-573.

62 Faizi S, Siddiqi H, Naz A, Bano S. Specific deuteration in patuletin and related flavonoids *via* keto–enol tautomerism: Solvent‐and temperature‐dependent ^1^H‐NMR Studies. Helv Chim Acta. 2010; 93(3) : 466-481.

63 Imran M, Mehmood R, Mughal UR, Ali B, Malik A. Vicarin, a new isoflavone from *Eremostachys vicaryi*. J Asian Nat Prod Res. 2012; 14: 293-296.

64 Aldrich Library of ^13^C and ^1^H FT NMR Spectra, 1992; 2, 1311B

65 Masuda T, Takasugi M, Anetai M. Psoralen and other linear furanocoumarins as phytoalexins in *Glehnia littoralis*. Phytochemistry. 1998; 47(1) : 13-16.

66 Sattar A, Ashraf M, Bhatty MK, Chisti NH. Beta cyclolavandulic acid and seselin in the essential oil of *Carum roxburghianum*. Phytochemistry 1978; 17: 559-560.

67 Bhatt Mehul, K, Dholwani Kishor K, Saluja Ajay K. (2011). Isolation and structure elucidation of scopoletin from *Ipomoea reniformis* (Convolvulaceae). J Appl Pharm Sci. 2011; 1(05): 138-144.

68 El-Demerdash A, Dawidar AM, Keshk EM, Abdel-Mogib M. Coumarins from *Cynanchum acutum*. Rev Latinoam Quím. 2009; 37: 65-69.

69 Rao PS, Asheervadam Y, Khaleelullah MD, Rao NS, Murray RDH. Hymexelsin, an apiose-containing scopoletin glycoside from the stem bark of *Hymenodictyon excelsum*. J Nat Prod. 1988; 51(5): 959-961.

70 Kerr KM, Kook AM, Davis PJ. High field and 2D-NMR studies with the aporphine alkaloid Glaucine. J Nat Prod. 1986; 49(4): 576–582.

71 Arafa AM, Mohamed MES, Eldahmy SI. The aerial parts of yellow horn poppy (*Glaucium flavum* Cr.) growing in Egypt: Isoquinoline alkaloids and biological activities. Int J Pharm Sci Res. 2016; 8(5): 323-332.

72 Alamzeb M, Khan MR, Mamoon-Ur-Rashid, Ali S, Khan AA. (2015). Isolation, structure elucidation and enzyme inhibition studies of a new hydroxy ester and other compounds from *Berberis jaeschkeana* Schneid stem. Nat Prod Res. 2015; 29: 1664-1669.

73 Manikyam HK, Ramesh C, Poluri KM, Assad A. Microwave assisted subcritical water extraction of berberine hydrochloride from the roots of *Berberis aristata* using Harmony search algorithm. J Herb Med Res. 2017; 2:19.

74 Tripathi AN, Chauhan L, Thankachan PP, Barthwal R. Quantum chemical and nuclear magnetic resonance spectral studies on molecular properties and electronic structure of berberine and berberrubine. Magn Reson Chem. 2007; 45(8): 647-655.

75 Couch JF. Lupine Studies. VIII. The Alkaloids of Lupinus palmeri, S. Wats. J Am Chem Soc. 1934; 56(11): 2434–2436.

76 Omeje EO, Osadebe PO, Nworu CS, Nwodo JN, Obonga WO, Kawamura A, Esimone CO, Proksch P. A novel sesquiterpene acid and an alkaloid from leaves of the Eastern *Nigeria mistletoe*, *Loranthus micranthus* with potent immunostimulatory activity on C57BL6 mice splenocytes and CD69 molecule. Pharm Bio, 2011; 49:1271-1276.

77 Rycroft DS, Robins DJ, Sadler IH. Revised assignment of the ^1^H NMR spectrum of the quinolizidine alkaloid lupinine. Magn Reson Chem. 1992; 30(13): S15-S17.

78 Kim SM, Kim YS, Kim DW, Yang JW. Transition metal-free, NaOt Bu-O2-mediated one-pot cascade oxidation of allylic alcohols to α, β-unsaturated carboxylic acids. Green Chem. 2012; 14(11): 2996-2998.

79 Shabbir S, Fatima I, Inamullah F, Mughal UR, Khan S, Kazmi MH, Malik A, Tareen RB, Abbas T. Brahin, a new lipoxygenase inhibiting triterpene from *Spiraea brahuica*. Chem Nat Compd. 2016; 52(6):1044-1046.

80 Sarria-Villa RA, Gallo-Corredor JA, Páez MI. Isolation of catechin and gallic acid from colombian bark of Pinus patula. Chem Sci J. 2017; 8: 174.

81 Mehta BK, Savita Sharma K, Dubey A. 4-Ethylgallic acid from two Mimosa species. Phytochemistry. 1988; 27(9): 3004–3005.

82 Chang SW, Kim KH, Lee IK, Choi SU, Ryu SY, Lee KR. Phytochemical constituents of *Bistorta manshuriensis*. Nat Prod Sci. 2009; 15(4): 234-240.

83 Waller CW, Gisvold O. A phytochemical investigation of *Larrea divaricata* Cav. J Am Pharm Assoc. 1945; 34(3): 78-81.

84 Son JK, Lee SH, Nagarapu L, Jahng YD. A simple synthesis of nordihydroguaiaretic acid and its analogues. Bull. Korean Chem. Soc. 2005; 26(7): 1117-1120.

85 Page JO. Extraction and purification of nordihydroguaiaretic acid. Anal Chem. 1951; 23(2): 296-298.

86 Agüero MB, Svetaz L, Sánchez M, Luna L, Lima B, López ML, Zacchino S, Palermo J, Wunderlin D, Feresin GE, Tapia A. Argentinean Andean propolis associated with the medicinal plant *Larrea nitida* Cav. (Zygophyllaceae). HPLC–MS and GC–MS characterization and antifungal activity. Food Chem Toxicol, 2011; 49(9): 1970-1978.
